# Reduced microRNA-744 expression in mast cell-derived exosomes triggers epithelial cell ferroptosis in acute respiratory distress syndrome

**DOI:** 10.1016/j.redox.2024.103387

**Published:** 2024-10-03

**Authors:** Xiaobin Fang, Fei Gao, Ling Zheng, Fu-Shan Xue, Tao Zhu, Xiaochun Zheng

**Affiliations:** a Department of Anesthesiology/Critical Care Medicine, Shengli Clinical Medical College of Fujian Medical University, Fujian Provincial Key Laboratory of Critical Care Medicine, Fujian Provincial Hospital, Fuzhou University Affiliated Provincial Hospital, Fuzhou, Fujian, China; b Department of Anesthesiology, West China Hospital, Sichuan University & The Research Unit of West China (2018RU012), Chinese Academy of Medical Science, Chengdu, Sichuan, China; c Department of Anesthesiology, Fujian Provincial Hospital, Shengli Clinical Medical College of Fujian Medical University & Fujian Emergency Medical Center, Fujian Provincial Key Laboratory of Emergency Medicine, Fujian Provincial Key Laboratory of Critical Medicine, Fujian Provincial Co-constructed Laboratory of “Belt and Road,”, Fuzhou, Fujian, China

**Keywords:** Acute lung injury, Exosome, Ferroptosis, Mast cell, microRNA

## Abstract

Acute respiratory distress syndrome (ARDS) is a critical disorder characterized by immune-related damage to epithelial cells; however, its underlying mechanism remains elusive. This study investigated the effects of alterations in microRNA (miRNA) expression in mast cell-derived exosomes on human bronchial epithelial (HBE) cells and ARDS development in cellular and mouse models challenged with lipopolysaccharide. Lipopolysaccharide-treated mast cell-derived exosomes reduced glutathione peroxidase 4 (*GPX4*) expression and increased long-chain acyl-CoA synthetase 4 (*ACSL4*), 15-lipoxygenase (*ALOX15*), and inflammatory mediator levels in HBE cells. miRNA sequencing revealed a reduction in mast cell-derived exosomal miR-744 levels, which was associated with the regulation of ACSL4, ALOX15, and GPX4 expression. This downregulation of exosomal miR-744 expression reduced miR-744 levels and promoted ferroptosis in HBE cells, whereas the experimental upregulation of miR-744 reversed these adverse effects. Down-regulation of miR-744 induced the expression of markers for ferroptosis and inflammation in HBE cells and promoted pulmonary ferroptosis, inflammation, and injury in LPS-stimulated mice. *In vivo*, treatment with *ACSL4*, *ALOX15*, and *GPX4* inhibitors mitigated these effects, and experimental miR-744 expression rescued the lipopolysaccharide-induced changes in HBE cells and mouse lungs. Notably, miR-744 levels were reduced in the plasma and exosomes of patients with ARDS. We concluded that decreased mast cell-derived exosomal miR-744 levels trigger epithelial cell ferroptosis, promoting lung inflammation and damage in ARDS. This study provides new mechanistic insights into the development and sustained pulmonary damage associated with ARDS and highlights potential therapeutic strategies.

## Highlights

• Mast cell-derived exosomes trigger epithelial ferroptosis in LPS-induced ARDS.
• Reduced miR-744 in mast cell exosomes induces ferroptosis and epithelial injury.
• MiR-744 downregulation induces ferroptosis and lung injury via GPX4, ACSL4, ALOX15.
• MiR-744 reduces LPS-induced epithelial ferroptosis and mouse lung injury.
• MiR-744 is significantly lower in the plasma and exosomes of patients with ARDS.

## Graphical abstract

**Figure:**
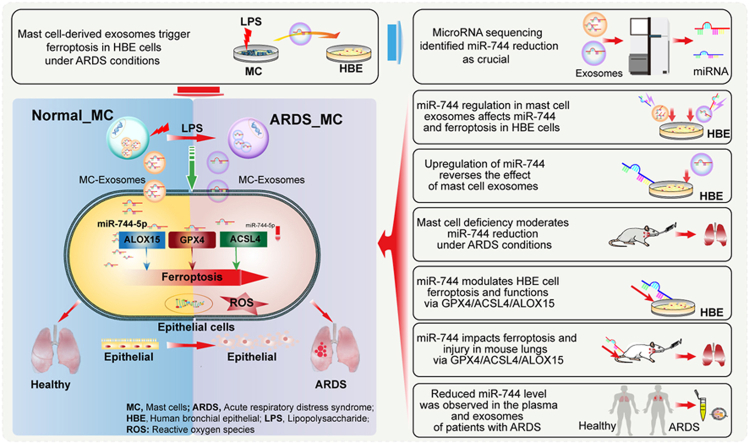

## 1 Introduction

Acute respiratory distress syndrome (ARDS), characterized by extensive inflammation and damage to both epithelial and endothelial cells, is a major disorder with mortality rates of >40 % in severe cases [1,2]. Damage to bronchial epithelial cells, often triggered by pathogen activities, plays a critical role in the progression of ARDS [3]. Pathogens can exacerbate the progression of ARDS by directly injuring epithelial cells and activating immune cells, which continues to adversely affect epithelial cell function even after clearance of the initial pathogens [4]. However, the exact mechanisms behind this sustained damage remain unclear.

Mast cells—key mediators of immune responses—have been implicated in the development of ARDS [5,6]. The interaction between mast and epithelial cells is essential for maintaining epithelial cell function and homeostasis [7]. Exosomes (extracellular vesicles with a diameter of 30–150 nm) [8,9] and exosomal microRNAs (miRNAs, 18–25-nucleotide-long non-coding RNAs) play crucial roles in these interactions [10]. The composition and abundance of exosomal miRNAs are altered under pathological conditions, such as ARDS, contributing to disease progression [11]. Lipopolysaccharide (LPS), a component of gram-negative bacterial cell membranes and a key pathogenic factor for ARDS [12], can alter the exosomal miRNA profiles of mast cells [13], potentially exacerbating epithelial cell injury.

In ARDS, epithelial cell injury occurs through various cell death mechanisms [14], including ferroptosis—an important iron-driven cell death process caused by lipid peroxidation [15]. The pharmacological inhibition of ferroptosis has shown potential in mitigating ARDS [16]. Ferroptosis involves key genes such as glutathione peroxidase 4 (*GPX4*), which neutralizes lipid peroxides [17]; long-chain acyl-CoA synthetase 4 (*ACSL4*), which promotes lipid peroxidation [18]; and 15-lipoxygenase (*ALOX15*), which oxidizes unsaturated lipids [19]. miRNAs, such as miR-130b, can regulate the expression of these genes [20]. In ARDS, mast cell-derived exosomal miRNAs can induce ferroptosis in epithelial cells by targeting *GPX4*, *ACSL4*, and *ALOX15*, thereby exacerbating ARDS development. However, the connection between the altered profiles of mast cell-derived exosomal miRNA and epithelial ferroptosis in ARDS remains unclear.

In this study, we aimed to determine the alterations in miRNA profiles in mast cell-derived exosomes under LPS-induced ARDS conditions and assess their roles in epithelial cell ferroptosis and disease pathogenesis. Understanding the mechanistic roles of specific miRNAs will improve our understanding of the pathophysiology of ARDS and assist in identifying new molecular targets for its treatment.

## 2 Materials and methods

### 2.1 Experimental design and ethical considerations

The various reagents, animals, cell lines, and culture conditions used in this study are provided here and in the Supplementary Material. Figure S1 outlines the experimental workflow. Th e Fujian Provincial Hospital's Experimental Animal Welfare Ethics Committee approved all animal care and research protocols (Certificate Number: FPH.PZ.20230506[0002]), which conformed to the National Institutes of Health's guidelines for the use of experimental animals. The reagents used in this study are listed in Table S1.

### 2.2 Cell culture and exosome isolation and characterization

The human mast cell (HMC)-1 and human bronchial epithelial (HBE) cell lines, sourced from the Shanghai Cell Bank (Shanghai, China), were cultured in RPMI 1640 medium. Subsequently, these cells were treated with either phosphate-buffered saline (PBS) or LPS. Exosomes were isolated following treatment and quantified using a Pierce™ BCA Protein Assay kit (Thermo Fisher Scientific, Waltham, MA, USA). The exosomes were characterized using transmission electron microscopy (TEM), nanoparticle tracking analysis (NTA), and western blotting. Detailed protocols for these techniques are provided in the Supplementary Text.

### 2.3 Exosome labeling, uptake study, and co-culture stimulation of HBE cells

Hypothesizing that exosomes facilitate functional changes, we treated HBE cells with conditioned medium from HMC-1 cells stimulated with PBS (PBS-MC-CM) or LPS (LPS-MC-CM). LPS was applied at a concentration of 1 μg/mL [21,22]. The uptake of 1,1′-dioctadecyl-3,3,3′,3′-tetramethylindocarbocyanine perchlorate (Dil)-labeled exosomes by HBE cells was analyzed using fluorescence microscopy. The HBE cells were then exposed to exosomes derived from HMC-1 cells (following PBS or LPS stimulation), with or without additional LPS treatment, to assess the effect of mast cell-derived exosomes on HBE cell functionality.

### 2.4 Exosomal miRNA sequencing and bioinformatic analysis

Exosomal miRNA sequencing was conducted in collaboration with Kangce Technology (Wuhan, China, Project No: KC2023–H0149). Sequence quality checks included read distribution across chromosomes using Circos visualization as well as Q20 (error rates <1 %) and Q30 (error rates <0.1 %) metrics. Differentially expressed miRNAs (DE_miRs) were identified using R 4.2.3 (The R Foundation for Statistical Computing, Vienna, Austria) and EdgeR software (Bioconductor, Boston, MA, USA), followed by principal component analysis (PCA), heatmap analysis, and initial screening using volcano plots (*P* < 0.05 and |log(fold-change)| > 1.5). To identify key miRNAs involved in in ferroptosis, the De_miRs associated with *ACSL4*, *ALOX15*, and *GPX4* regulation were identified using MultiR [20]. The sequencing data have been deposited in the GEO database.

### 2.5 Transfection of exosomes with miRNA mimics or antagonists

An exosome suspension mixed with miRNA solution was transferred to a 2 mm gap electroporation cuvette. Electroporation of exosomes was conducted using a NEPA21 electroporator (Nepa Gene, Chiba, Japan) set to 100 V, 5-ms pulse widths, and 2 pulses at an electrical resistance of 30–50 Ω. After electroporation, the samples were collected and stored for future use.

### 2.6 Transfection of HBE cells with miRNA mimics and antagonists

HBE cells were transfected with miR-negative control (NC), miR-744, or anta-miR-744. Following transfection, the cells were treated with specific inhibitors (PRGL493 for ACSL4, ALOX15-IN-2 for ALOX15, and GPX4-IN-3 for GPX4) to study the changes in protein expression. A dual-luciferase reporter assay was performed to assess the regulation of miR-744 expression by GPX4, ACSL4, and ALOX15. The cell responses and changes in ferroptosis were assessed using a labile iron pool (LIP), TEM, reactive oxygen species (ROS), cell counting kit-8 (CCK-8), Transwell, flow cytometry, and scratch assays. Table S2 lists the miRNA sequences used.

### 2.7 Animals and experimental procedures

Male BALB/c and mast cell-deficient C57BL/6-Kit^W-sh/W−sh^ mice (20–22 g, 8–12 weeks, details in Supplementary Materials) were used. We first examined the effects of anta-miR-744 on lung function and ACSL4, ALOX15, and GPX4 regulation. We then assessed the protective role of miR-744 against LPS-induced ARDS and sepsis. Mice were administered PBS, miR-NC, or miR-744 as described in previous reports [23], followed by LPS to induce lung injury or sepsis. Randomized group assignment was performed using R software. To explore the interaction between mast cells and ARDS-related ferroptosis, inflammation, and miR-744 expression, an ARDS model was established in mast cell-deficient (MC−/−) and wild-type C57BL/6 mice via intratracheal (50 μL, 1 μg/μL) and intraperitoneal (50 μL, 3 μg/μL) administration of LPS to induce lung injury and sepsis, respectively [24,25]. After 24 h, mice were euthanized with an overdose of sodium pentobarbital (100 mg/kg, intraperitoneally) for sample collection.

### 2.8 In vivo epithelial cell isolation

We examined the in-vivo effects of anta-miR-744 on epithelial cells in single-cell isolation experiments. Briefly, mice were intratracheally injected with anta-miR-744 or miR-NC and euthanized 24 h post-injection. Lung tissues were collected for epithelial cell isolation as previously described [[26], [27], [28]]. Single-cell suspensions were prepared using a Lung Dissociation Kit (cat. no. 130-095-927; Miltenyi Biotec) following the manufacturer's instructions. After removing red blood cells, the remaining cells were co-incubated with cluster of differentiation (CD)45 (cat. no. 147711; BioLegend, San Diego, CA, USA) and CD326 (cat. no. 118213; BioLegend) antibodies. Subsequently, CD45^−^CD326^+^ epithelial cells were isolated using flow cytometry and subjected to biochemical analyses.

### 2.9 Clinical sample collection and analysis

The Fujian Provincial Hospital Ethics Committee approved the study protocol (Approval Number: K2023-03-035), which conformed to the principles outlined in the Declaration of Helsinki. Patient consent was obtained before participation. Clinical samples were obtained from six patients with ARDS who had a partial pressure of arterial oxygen to fractional inspired oxygen concentration PaO_2_/FiO_2_ ratio of <200 mmHg due to infection. Plasma and exosomes were extracted from 10 mL of peripheral blood for subsequent quantitative real-time polymerase chain reaction (qPCR) analysis. For initial validation, one sample from each of the six patients with ARDS was chosen, allowing for a subsequent larger-scale clinical trial.

### 2.10 qPCR, western blotting, enzyme-linked immunosorbent assay, and histological analysis

The mRNA and miRNA levels in the clinical blood samples were determined using qPCR and normalized to those of *18S RNA* and *U6*, respectively. The primers for qPCR are listed in Table S3. Protein levels of GPX4, ACSL4, and ALOX15 were determined through western blotting, and myeloperoxidase (MPO) levels in the lung tissues were measured using enzyme-linked immunosorbent assay (ELISA). Protein expression was quantified from the western blots using the ImageJ software (National Institutes of Health, Bethesda, MD, USA). Histopathological changes and miRNA distribution in the lung tissues were examined using hematoxylin and eosin (H&E) staining and fluorescence colocalization analysis. Lung injury scores were used to quantify pulmonary damage [21].

### 2.11 Statistical analysis

Normally distributed data are presented as the mean ± standard error of the mean. The Shapiro–Wilk test was used to assess data normality. Statistical analyses, including Student's *t-*test, one-way analysis of variance, and Dunnett's multiple comparison tests, were performed using R 4.2.3. Statistical significance was set at *P* < 0.05.

## 3 Results

### 3.1 Characterization of exosomes and their internalization in HBE cells

**Fig. 1:**
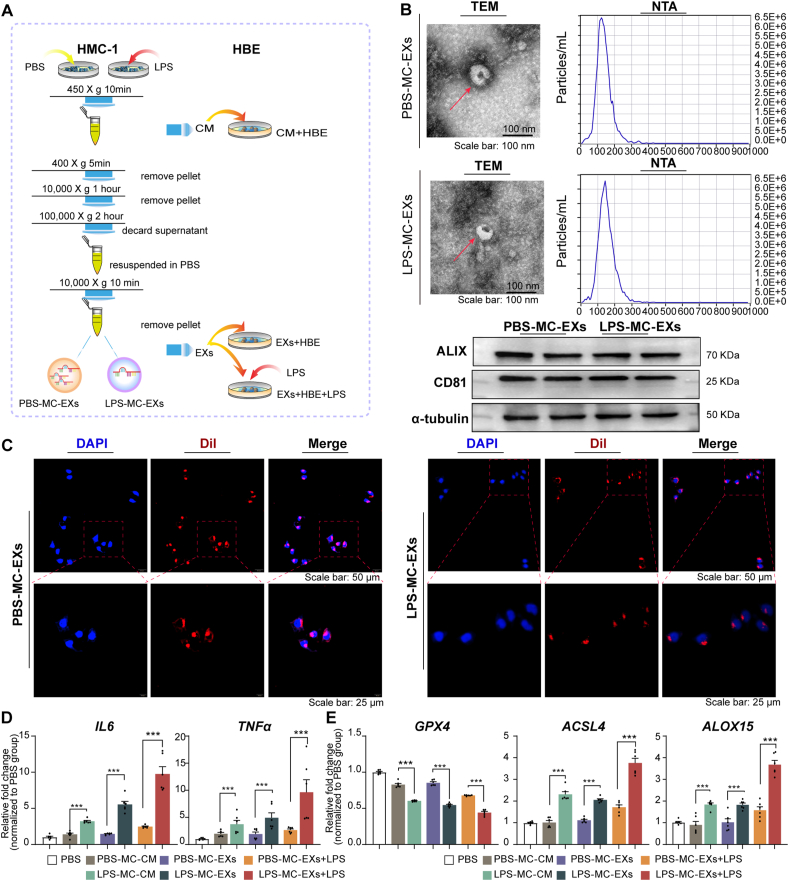
Exosomes from LPS-treated mast cells induced ferroptosis and inflammation in HBE cells. HBE cells were incubated with the conditioned medium of HMC-1 cells stimulated with LPS or PBS or exosomes, with or without additional LPS treatment. The experiments were conducted with three independent technical replicates. **(A)** Flowchart illustrating the experimental procedure. **(B)** Exosome characterization. Representative images for TEM (upper left), NTA (upper right), and western blots for the exosomal markers CD81 and ALIX (lower panel). **(C)** Representative images of the exosome uptake experiment. **(D)** qPCR results for *IL6* and *TNFa*. **(E)** qPCR results for *GPX4*, *ACSL4*, and *ALOX15*. ∗∗∗*P* < 0.001 (ANOVA, followed by multiple comparison tests). *ACSL4*, long-chain acyl-CoA synthetase 4; *ALOX15*, 15-lipoxygenase; *GPX4*, glutathione peroxidase 4; HBE, human bronchial epithelial; LPS, lipopolysaccharide; NTA, nanoparticle tracking analysis; PBS, phosphate-buffered saline; TEM, transmission electron microscopy; LPS-MC-CM, conditioned medium from HMC-1 cells stimulated with LPS; PBS-MC-CM, conditioned medium from HMC-1 cells stimulated with PBS; LPS-MC-EX, exosome from HMC-1 cells stimulated with LPS; PBS-MC-EX, exosome from HMC-1 cells stimulated with PBS.

Following the experimental procedure illustrated in Fig. 1A, TEM revealed morphologically distinct membrane-bound particles (exosomes) with a diameter of ∼200 nm (Fig. 1B). The NTA showed that the median diameters of exosomes from HMC-1 cells stimulated with PBS (PBS-MC-EXs) and LPS (LPS-MC-EXs) were 129.9 and 142.6 nm, respectively (Fig. 1B). PBS-MC-EXs and LPS-MC-EXs expressed CD81 and ALIX (Fig. 1B). The exosome uptake experiments revealed a distinct fluorescence signal in the cytoplasm of nearly all HBE cells after co-incubation with Dil-labeled exosomes, demonstrating successful exosome internalization (Fig. 1C).

### 3.2 LPS-treated mast cell-derived conditioned medium and exosomes induced HBE cell ferroptosis and inflammation

Compared with those co-incubated with PBS-MC-CM, HBE cells co-incubated with LPS-MC-CM exhibited elevated interleukin (IL) and tumor necrosis factor-alpha (TNF-α) expression (Fig. 1D). Additionally, *ACSL4* and *ALOX1*5 mRNA levels increased markedly, while those of *GPX4* decreased (Fig. 1E). Similarly, compared with those treated with PBS-MC-EXs, HBE cells treated with LPS-MC-EXs exhibited increased *IL6*, *TNFa*, *ACSL4*, and *ALOX1*5 mRNA expression and decreased *GPX4* mRNA expression (Fig. 1D and E).

Pre-treatment with LPS-MC-EXs followed by LPS enhanced *ACSL4*, *ALOX15*, *IL6*, and *TNFa* mRNA expression and reduced *GPX4* expression in HBE cells. These findings reflect the augmented susceptibility of HBE cells treated with LPS-MC-EXs to ferroptosis after LPS exposure (Fig. 1D and E).

### 3.3 Exosomal miRNA profiling identified miR-744 as a key regulator of ferroptosis-associated genes

**Fig. 2:**
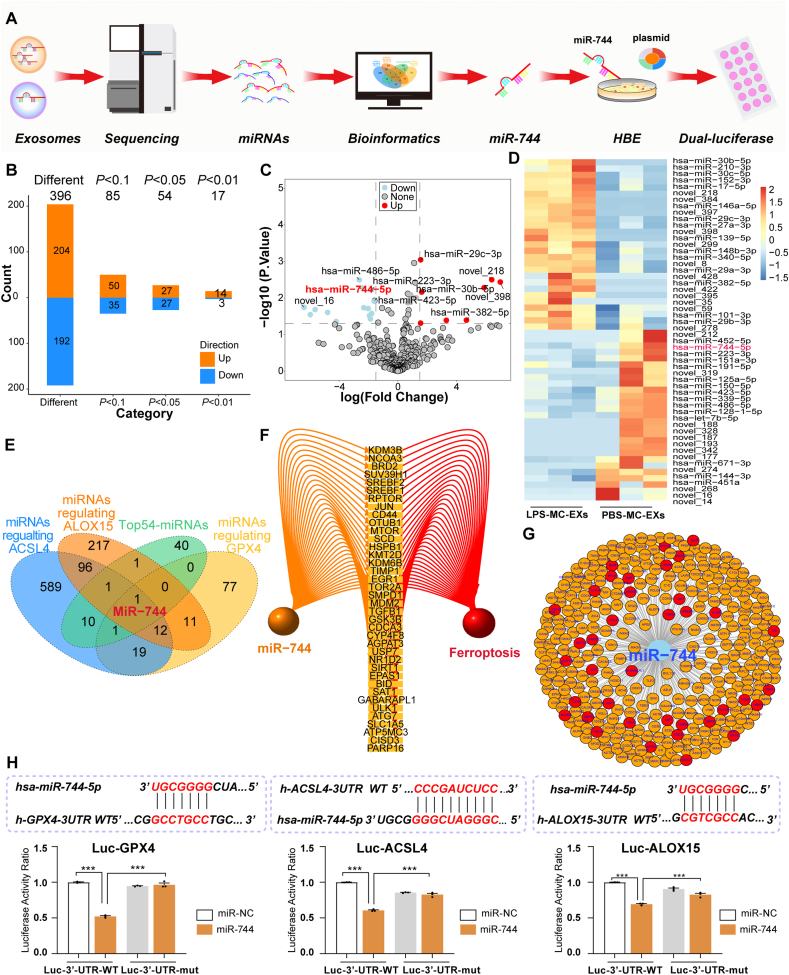
Screening and validation of miR-744 as a key regulator of ferroptosis in mast cell-derived exosomes. **(A)** Flowchart of the experimental procedure. HMC-1 cells were stimulated with LPS or PBS, and exosomes were collected for miRNA sequencing. Data were analyzed using R (4.4.1). **(B)** Exosomal miRNA sequencing revealed differential expression of miRNA in PBS-MC-EXs and LPS-MC-EXs. **(C)** Volcano plot of differentially expressed miRNAs. **(D)** Heatmap of the top 54 miRNAs (*P* < 0.05) in PBS-MC-EXs and LPS-MC-EXs. **(E)** Intersection analysis of top 54 miRNAs along with miRNAs regulating the *ACSL4*/*ALOX15*/*GPX4* axis. **(F)** Ferroptosis-associated genes whose expression is regulated by miR-744. **(G)** Target genes of miR-744 are highlighted with ferroptosis-associated genes in red. **(H)** Dual-luciferase assay results. *ACSL4*, long-chain acyl-CoA synthetase 4; *ALOX15*, 15-lipoxygenase; ARDS, acute respiratory distress syndrome; *GPX4*, glutathione peroxidase 4; HBE, human bronchial epithelial; LPS, lipopolysaccharide; LPS-MC-EX, exosome from HMC-1 cells stimulated with LPS; PBS-MC-EX, exosome from HMC-1 cells stimulated with PBS. (For interpretation of the references to colour in this figure legend, the reader is referred to the Web version of this article.)

The experimental workflow for identifying and validating miR-744 as a regulator of ferroptosis in mast cell-derived exosomes is depicted in Fig. 2A. Circos plots confirmed uniform read distribution (Fig. S2A) and sequencing quality was high (Q20 score >99 %, Q30 score >97 %; Fig. S2B). Exosomal miRNA sequencing revealed 396 DE_miRs between PBS-MC-EXs and LPS-MC-EXs (Fig. 2B). PCA revealed differences in miRNA profiles between the groups (Fig. S2C). Volcano plot analysis identified 14 upregulated and 23 downregulated miRNAs (Fig. 2C). Among the top 54 miRNAs with the most pronounced differential expression between the two groups (all *P* < 0.05, Fig. 2D), miR-744 ranked third in terms of significance. Intersection analysis between these miRNAs and those known to regulate the ACSL4/ALOX15/GPX4 axis revealed miR-744 as a key target (Fig. 2E). miR-744 was revealed to regulate several genes pivotal to ferroptosis (Fig. 2F and G). The dual-luciferase assay revealed that HBE cells transfected with miR-744 exhibited a considerable decrease in luciferase activity compared with miR–NC–treated cells, indicating that miR-744 regulates the expression of *ACSL4*, *GPX4*, and *ALOX15* (Fig. 2H).

### 3.4 Regulation of miR-744 in mast cell-derived exosomes affected miR-744 expression and ferroptosis in HBE cells

**Fig. 3:**
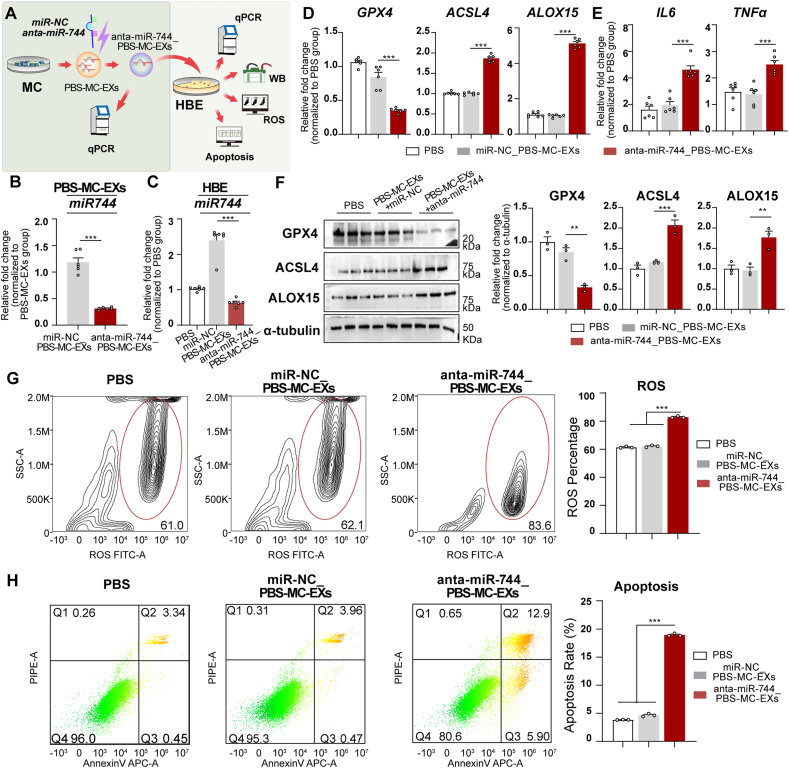
Downregulation of miR-744 in normal mast cell-derived exosomes reduced miR-744 expression and triggered ferroptosis in HBE cells. **(A)** Flowchart depicting the experimental design. PBS-MC-EXs were transfected with either a negative control microRNA (miR-NC_ PBS-MC-EXs) or anta-miR-744(anta-miR-744_ PBS-MC-EXs), and then incubated with HBE cells. The experiments were performed with three independent technical replicates. **(B)** qPCR results for miR-744 expression in PBS-MC-EXs after transfection with anta-miR-744. **(C–H)** Analysis of HBE cells after incubation with PBS-MC-EXs transfected with anta-miR-744. **(C)** qPCR results showing miR-744 expression. (**D)** qPCR results showing *GPX4*, *ACSL4*, and *ALOX15* expression. **(E)** qPCR results for *IL6* and *TNFa* expression. **(F)** Left panel: Representative western blots for GPX4, ACSL4, and ALOX15; Right panel: Blots were quantified using the ImageJ software. **(G)** Flow cytometry data for ROS, including representative images (left) and quantitative analysis (right). (**H)** Flow cytometry data for apoptosis, including representative images (left) and quantitative analysis (right). ∗*P* < 0.05, ∗∗*P* < 0.01, ∗∗∗*P* < 0.001 (ANOVA, followed by multiple comparison tests). *ACSL4*, long-chain acyl-CoA synthetase 4; *ALOX15*, 15-lipoxygenase; *GPX4*, glutathione peroxidase 4; HBE, human bronchial epithelial; LPS, lipopolysaccharide; miRNA, microRNA; qPCR, quantitative real-time PCR; PBS-MC-EX, exosome from HMC-1 cells stimulated with PBS; WB, Western blot.

Following the experimental procedure depicted in Fig. 3A, we confirmed the successful transfection of PBS-MC-EXs with anta-miR-744 based on a reduction in miR-744 expression (Fig. 3B). Co-incubation with anta-miR-744-transfected exosomes decreased miR-744 levels in HBE cells (*P* < 0.001, Fig. 3C). Compared with those incubated with miR–NC–transfected exosomes, HBE cells incubated with anta-miR-744-transfected exosomes exhibited increased *ACSL4*, *ALOX1*5, *IL6*, and *TNFa* mRNA levels and decreased *GPX4* mRNA levels (Fig. 3D and E). Western blot analysis supported these findings by demonstrating an increase in ACSL4 and ALOX15 levels and decrease in GPX4 levels (Fig. 3F). Flow cytometry revealed that anta-miR-744-transfected exosomes increased ROS (Fig. 3G) and apoptosis levels in HBE cells (Fig. 3H). These findings suggested that miR-744 downregulation in mast cell-derived exosomes reduced miR-744 expression and triggered ferroptosis and inflammation in HBE cells.

**Fig. 4:**
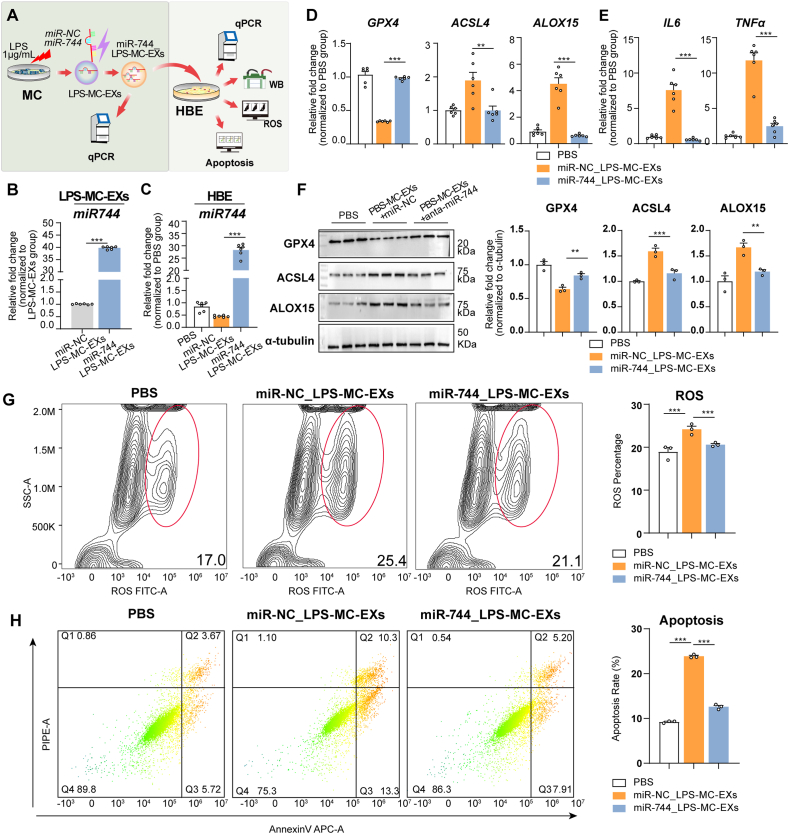
Upregulation of miR-744 expression in LPS-treated mast cell-derived exosomes increased miR-744 levels and mitigated ferroptosis in HBE cells. **(A)** Flowchart of experimental design. LPS-MC-EXs were transfected with either a miR-NC(miR-NC_ LPS-MC-EXs) or miR-744(miR-744_ LPS-MC-EXs), and then incubated with HBE cells. The experiments were performed with three independent technical replicates. **(B)** qPCR results for miR-744 expression in LPS-MC-EXs after transfection with miR-744. **(C–H)** Analysis of HBE cells after incubation with LPS-MC-EXs transfected with miR-744. **(C)** qPCR results showing miR-744 expression. (**D)** qPCR results for *GPX4*, *ACSL4*, and *ALOX15* expression. **(E)** qPCR results for *IL6* and *TNFa* expression. **(F)** Left panel: Representative western blots for *GPX4*, *ACSL4*, and *ALOX15*; Right panel: Blots were quantified using ImageJ. **(G)** Flow cytometry data for ROS, including representative images (left) and quantitative analysis (right). (**H)** Flow cytometry data for apoptosis, including representative images (left) and quantitative analysis (right). ∗*P* < 0.05, ∗∗*P* < 0.01, ∗∗∗*P* < 0.001 (ANOVA, followed by multiple comparison tests). *ACSL4*, long-chain acyl-CoA synthetase 4; *ALOX15*, 15-lipoxygenase; *GPX4*, glutathione peroxidase 4; HBE, human bronchial epithelial; LPS, lipopolysaccharide; miRNA, microRNA; qPCR, quantitative real-time PCR; LPS-MC-EX, exosome from HMC-1 cells stimulated with LPS; WB, Western blot.

Following the procedure shown in Fig. 4A, increased miR-744 expression in LPS-MC-EXs confirmed successful transfection (Fig. 4B). Co-incubation with LPS-MC-EXs transfected with miR-NC decreased miR-744 levels, whereas co-incubation with LPS-MC-EXs transfected with miR-744 increased miR-744 levels in HBE cells (*P* < 0.001, Fig. 4C). HBE cells co-incubated with LPS-MC-EXs transfected with miR-NC exhibited increased ACSL4, ALOX15, IL-6, and TNF-α expression and decreased GPX4 expression (qPCR results in Fig. 4D and E; western blots in Fig. 4F), as well as increased ROS (Fig. 4G) and apoptosis levels (Fig. 4H). However, co-incubation with LPS-MC-EXs transfected with miR-744 attenuated these alterations (Fig. 4D–H). These findings suggested that the upregulation of miR-744 in LPS-MC-EXs alleviated LPS-MC-EX-induced ferroptosis and inflammation in HBE cells.

### 3.5 Upregulation of miR-744 expression in HBE cells reversed the effect of LPS-MC-EXs

**Fig. 5:**
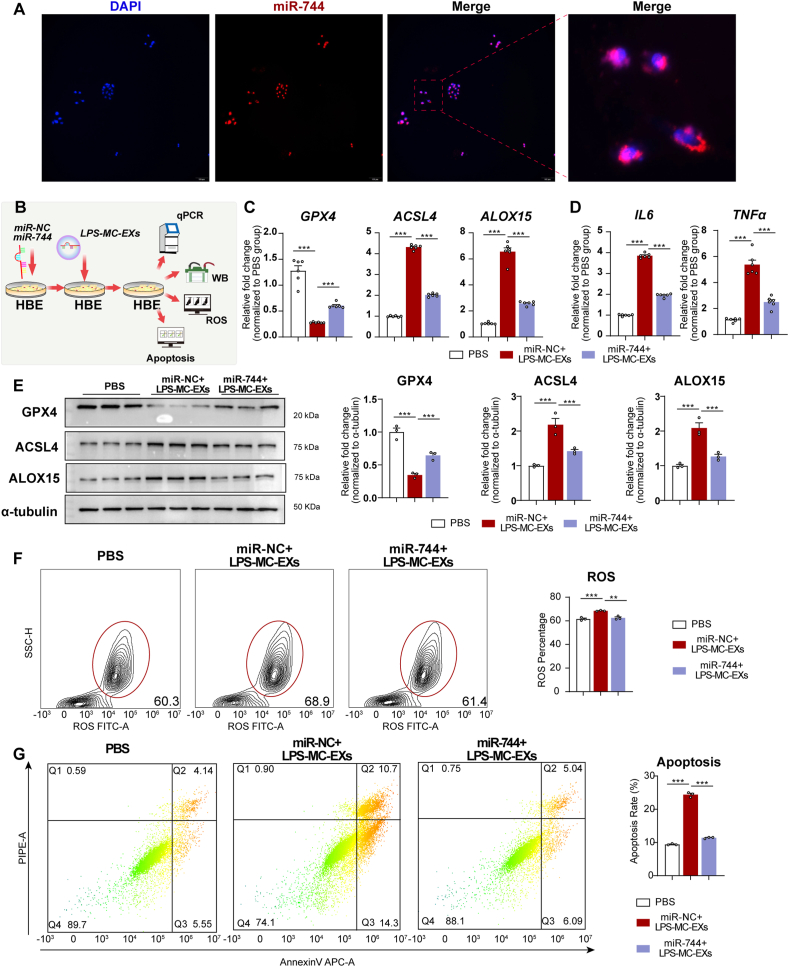
Upregulation of miR-744 in HBE cells reduced the effect of LPS-MC-EXs. **(A)** Representative images of miR-744 transfection experiment. HBE cells were transfected with miR-NC or miR-744 and then incubated with LPS-MC-EXs(miR-NC + LPS-MC-EXs or miR-744+LPS-MC-EXs). The experiments were performed with three independent technical replicates. **(B)** Flowchart of experimental procedure. HBE cells were transfected with miR-744 and then incubated with LPS-MC-EXs. **(C)** qPCR results for *GPX4*, *ACSL4*, and *ALOX15* expression. **(D)** qPCR results for *IL6* and *TNFa* expression. **(E)** Left panel: Representative western blots for *GPX4*, *ACSL4*, and *ALOX15*; Right panel: Blots were quantified using ImageJ. **(F)** Flow cytometry data for ROS, including representative images (left) and quantitative analysis (right). **(G)** Flow cytometry data for apoptosis, including representative images (left) and quantitative analysis (right). ∗*P* < 0.05, ∗∗∗*P* < 0.001 (ANOVA, followed by multiple comparison tests). *ACSL4*, long-chain acyl-CoA synthetase 4; *ALOX15*, 15-lipoxygenase; *GPX4*, glutathione peroxidase 4; HBE, human bronchial epithelial; LIP, labile iron pool; PBS, phosphate-buffered saline; TEM, transmission electron microscopy; ROS, reactive oxygen species.

Fluorescence imaging confirmed miR-744 transfection, showing red fluorescence in nearly all nuclei of HBE cells (Fig. 5A). Following the procedure depicted in Fig. 5B, upregulation of miR-744 in HBE cells followed by LPS-MC-EX exposure increased the mRNA and protein levels of GPX4 and decreased those of ACSL4, ALOX15, IL-6, and TNF-α (qPCR results in Fig. 5C and D; western blots in Fig. 5E), as well as reduced ROS (Fig. 5F) and apoptosis levels (Fig. 5G). These findings suggested that reduced miR-744 expression in HBE cells is crucial for initiating ferroptosis and inflammation.

### 3.6 Mast cell-deficient C57BL/6-Kit^W-sh/W−sh^ mice exhibited slightly reduced miR-744 expression and milder pulmonary inflammation and ferroptosis in ARDS

In the LPS-induced ARDS models (Fig. S3A), mast cell-deficient C57BL/6-Kit^W-sh/W−sh^ mice exhibited a marginal decrease in miR-744 expression compared to the wild-type controls (Fig. S3B), highlighting the regulatory role of mast cells in mediating LPS-induced miR-744 expression. C57BL/6-Kit^W-sh/W−sh^ mice also displayed attenuated pulmonary responses, as evidenced by the relatively low increase in the levels of inflammatory markers such as IL-6, TNF-α, and MPO; a milder upregulation of ACSL4 and ALOX15 expression; and a more modest decrease in GPX4 expression. These findings were supported by the qPCR (Figs. S3C and D) and ELISA results (Figs. S3E–G).

### 3.7 Anta-miR-744 transfection induced HBE cell ferroptosis via GPX4, ACSL4, and ALOX15

**Fig. 6:**
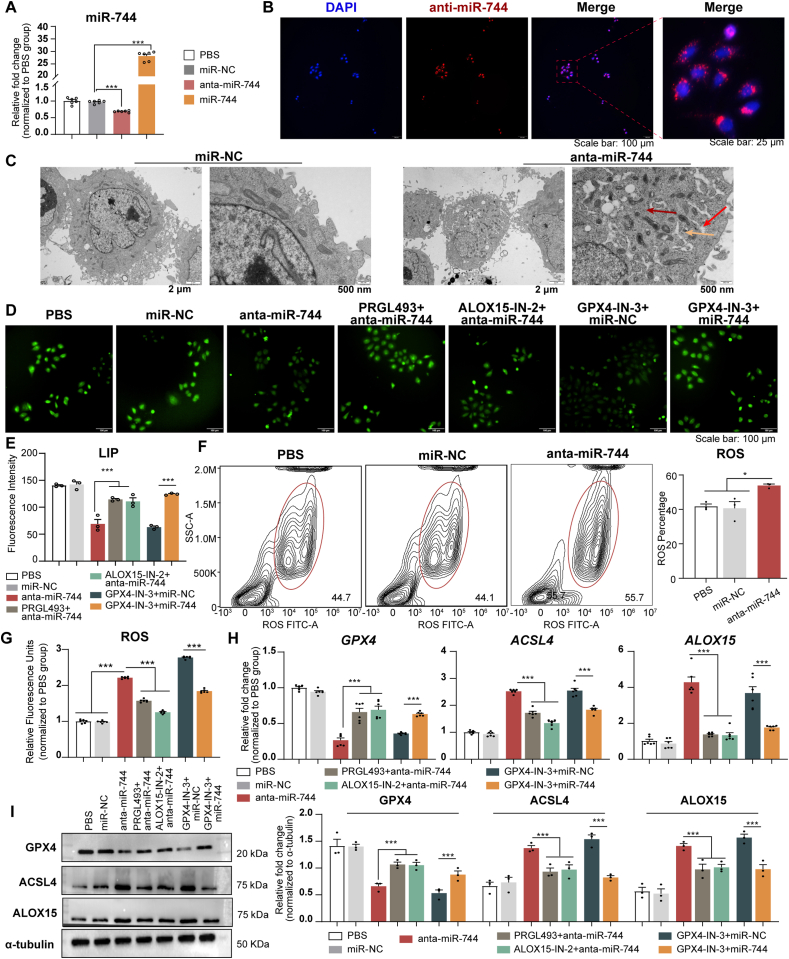
Anta-miR-744 transfection-induced HBE ferroptosis was mitigated by GPX4, ACSL4, and ALOX15 inhibitors. **(A)** qPCR results for miR-744 expression in HBE cells after transfection with miR-NC, miR-744, or anta-miR-744. **(B)** Representative images of anta-miR-744 transfection experiment. Representative images of miR-744 transfection experiment are shown in Fig. S4A. **(C–I)** HBE cells were analyzed after transfection with anta-miR-744, with or without the corresponding inhibitors. The experiments were performed with three independent technical replicates. **(C)**TEM results. Red arrows: mitochondrial shrinkage and denser membranes; yellow arrows: widened endoplasmic reticulum; dark-red arrows: increased number of vesicles. **(D)** Representative LIP images; **(E)** LIP images quantified using ImageJ. **(F)** Flow cytometry data for ROS, including representative images (left) and quantitative analysis (right). **(G)** Immunofluorescence assay results for ROS levels. **(H)** qPCR results for *GPX4*, *ACSL4*, and *ALOX15* expression. **(I)** Left panel: Representative western blots for GPX4, ACSL4, and ALOX15; Right panel: Blots were quantified using ImageJ. ∗*P* < 0.05, ∗∗∗*P* < 0.001 (ANOVA, followed by multiple comparison tests). *ACSL4*, long-chain acyl-CoA synthetase 4; *ALOX15*, 15-lipoxygenase; *GPX4*, glutathione peroxidase 4; HBE, human bronchial epithelial; LIP, labile iron pool; PBS, phosphate-buffered saline; TEM, transmission electron microscopy; ROS, reactive oxygen species. (For interpretation of the references to colour in this figure legend, the reader is referred to the Web version of this article.)

Transfection of HBE cells with anta-miR-744 resulted in reduced miR-744 levels, whereas transfection with miR-744 led to increased miR-744 levels (both *P* < 0.001, Fig. 6A). Fluorescence imaging confirmed efficient transfection, showing red fluorescence around nearly all HBE cell nuclei (Fig. 6B). Knockdown of miR-744 in HBE cells resulted in mitochondrial shrinkage, increased membrane density, cristae rupture, and cytoplasmic loosening, with vacuolation in certain areas (Fig. 6C). LIP analysis demonstrated a reduction in LIP levels following miR-744 knockdown, indicating increased cellular Fe^2+^ levels (Fig. 6D and E). Flow cytometry and immunofluorescence assay indicated that anta-miR-744 increased ROS levels, which were modulated by ACSL4 and ALOX15 inhibitors (Fig. 6F and G). qPCR analysis revealed elevated *ACSL4* and *ALOX1*5 mRNA levels but reduced *GPX4* mRNA levels in anta-miR-744-transfected HBE cells. These changes were neutralized by ACSL4 and ALOX15 inhibitors (Fig. 6H). Western blot analysis confirmed the influence of anta-miR-744 and its inhibitors on the expression of ACSL4, ALOX15, and GPX4 (Fig. 6I).

Treatment with the GPX4 inhibitor resulted in increased ROS, ACSL4, and ALOX15 levels and decreased GPX4 levels in HBE cells. However, miR-744 overexpression reversed these effects (Fig. 6F–I). These findings indicated that anta-miR-744 promotes ROS production and ferroptosis in HBE cells through the regulation of GPX4, ACSL4, and ALOX15.

### 3.8 Anta-miR-744 transfection modulated HBE cell function via GPX4, ACSL4, and ALOX15

**Fig. 7:**
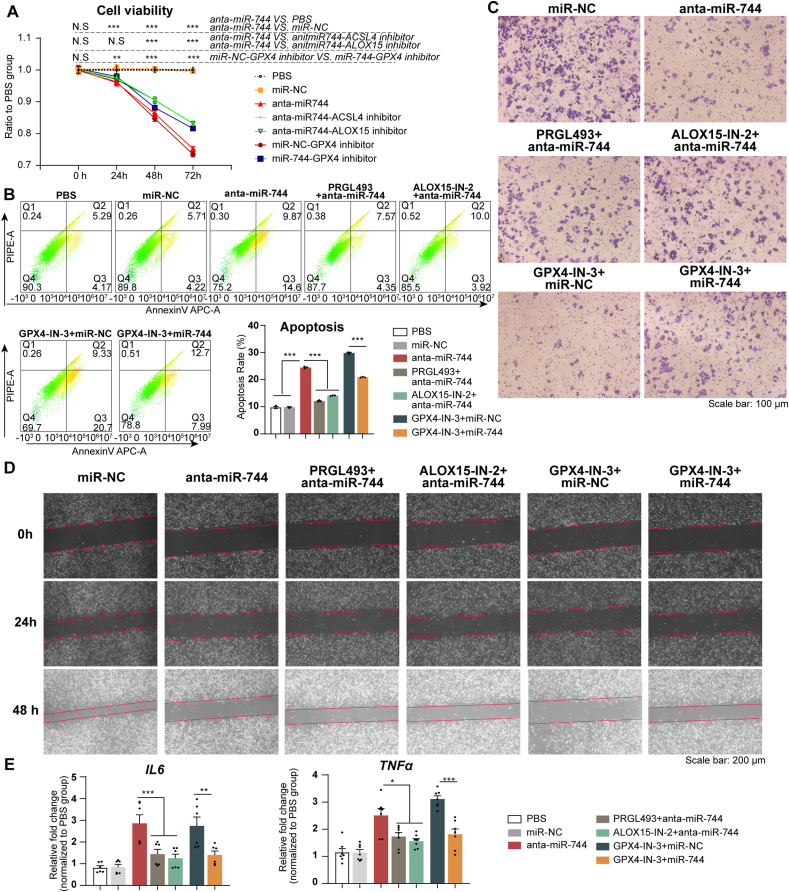
Anta-miR-744 transfection affected HBE cell viability, apoptosis, and migration via GPX4, ACSL4, and ALOX15 regulation. HBE cells were analyzed after transfection with anta-miR-744, with or without the corresponding inhibitors. The experiments were performed with three independent technical replicates. **(A)** CCK-8 assay results. **(B)** Flow cytometry results. Left panel: Representative flow cytometry images; Right panel: Quantification of flow cytometry images. **(C)** Representative images of Transwell migration assays. **(D)** Representative images of scratch assay. **(E)** qPCR results for *IL6* and *TNFa* expression. ∗*P* < 0.05, ∗∗*P* < 0.01, ∗∗∗*P* < 0.001 (ANOVA, followed by multiple comparison tests). *ACSL4*, long-chain acyl-CoA synthetase 4; *ALOX15*, 15-lipoxygenase; *GPX4*, glutathione peroxidase 4; HBE, human bronchial epithelial; miRNA, microRNA.

CCK-8 assays and flow cytometry demonstrated that the transfection of HBE cells with anta-miR-744 decreased cell viability and increased apoptosis levels. These negative effects were mitigated by ACSL4-and ALOX15-inhibitor treatment (Fig. 7A and B). Migration assays, including the Transwell and scratch assays, revealed that anta-miR-744 hindered the migration of HBE cells. However, the ACSL4 and ALOX15 inhibitors effectively reversed this impairment (Fig. 7C and D). qPCR analyses revealed that the elevated levels of *IL6* and *TNFa* induced by anta-miR-744 transfection were neutralized by treatment with the ACSL4 and ALOX15 inhibitors (Fig. 7E).

In contrast, treatment with the GPX4 inhibitor reduced cell migration and viability, and increased apoptosis and inflammation levels in HBE cells (Fig. 7A–E). These detrimental effects were reversed by miR-744 overexpression. These findings suggested that the downregulation of miR-744 compromises the viability, proliferation, and migration abilities of HBE cells via the regulation of GPX4, ACSL4, and ALOX15 expression.

### 3.9 Intratracheal administration of anta-miR-744 modulates ferroptosis, inflammation, and injury in mouse lungs via GPX4, ACSL4, and ALOX15

**Fig. 8:**
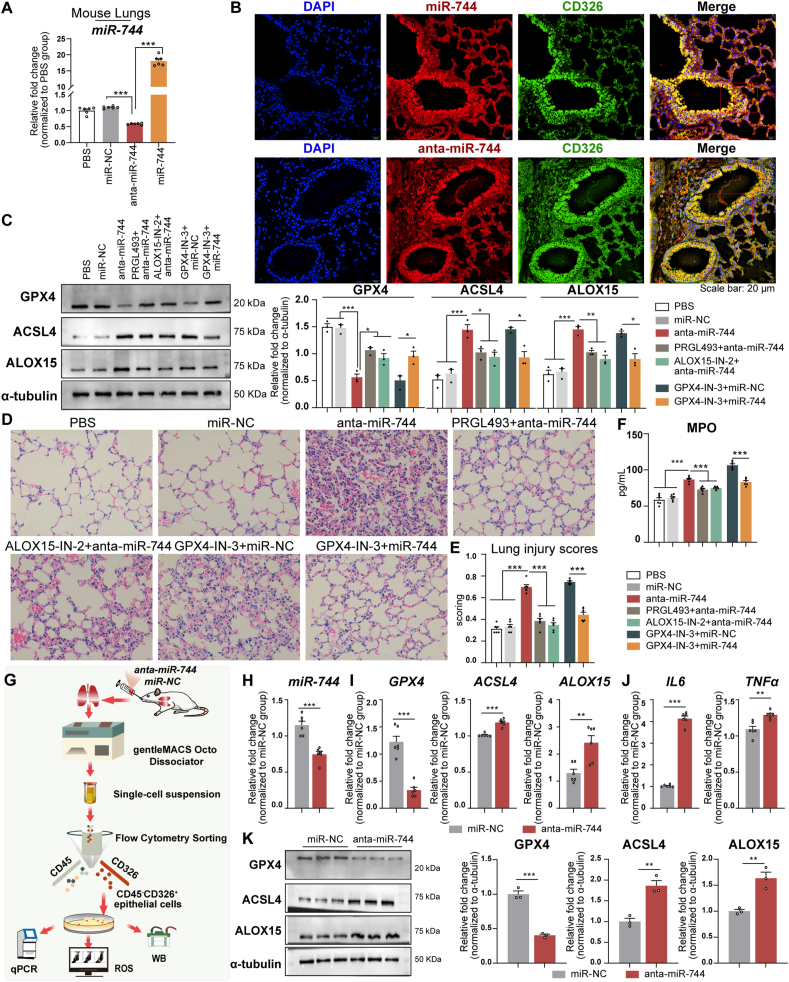
Intratracheal administration of anta-miR-744 modulated ferroptosis, inflammation, and injury in mouse lungs via GPX4, ACSL4, and ALOX15 regulation. **(A**–**F)** BALB/c mice were administered miR-744 or anta-miR-744 intratracheally, and lungs were analyzed after 24 h (*n* = 6 mice/group). **(A)** miR-744 expression in lung tissue. **(B)** Fluorescence microscopy showing epithelial uptake of miR-744/anta-miR-744. **(C)** Left panel: Representative western blots for GPX4, ACSL4, and ALOX15; Right panel: Blots quantified using ImageJ. **(D)** Representative H&E staining images. **(E)** H&E staining results were quantified in a blinded manner to determine the lung injury scores. **(F)** ELISA for myeloperoxidase activity. **(G)** Flowchart of experiments using pulmonary epithelial cells. **(H)** qPCR results for miR-744 in isolated pulmonary epithelial cells. **(I)** qPCR results for *GPX4*, *ACSL4*, and *ALOX15* expression. **(J)** qPCR results for *IL6* and *TNFa* expression. **(K)** Left panel: Representative western blots for *GPX4*, *ACSL4*, and *ALOX15*; Right panel: Blots quantified using ImageJ. **(A**–**F)** ∗*P* < 0.05, ∗∗*P* < 0.01, ∗∗∗*P* < 0.001 (ANOVA, followed by multiple comparisons test); **(H–K)** ∗*P* < 0.05, ∗∗*P* < 0.01, ∗∗∗*P* < 0.001 (Student's *t*-test). *ACSL4*, long-chain acyl-CoA synthetase 4; *ALOX15*, 15-lipoxygenase; *GPX4*, glutathione peroxidase 4; HBE, human bronchial epithelial; H&E, hematoxylin and eosin; miRNA, microRNA.

Intratracheal administration of anta-miR-744 decreased miR-744 levels in the lung tissue of BALB/c mice, whereas administration of miR-744 increased these levels compared with those in the miR–NC–treated mice (*P* < 0.001, Fig. 8A). Fluorescent-labeled miR-744 or anta-miR-744 was administered intratracheally, and fluorescence microscopy revealed the colocalization of red fluorescence with green CD326 and blue DAPI signals (Fig. 8B), confirming epithelial cell uptake. Western blot analyses demonstrated that anta-miR-744 treatment increased pulmonary ACSL4 and ALOX15 levels while decreasing GPX4 levels (Fig. 8C). These changes were reversed by pre-treatment with ACSL4 and ALOX15 inhibitors. Anta-miR-744 altered the expression of *ACSL4*, *ALOX15*, *GPX4*, *IL6*, and *TNFa* mRNA, which was also reversed by pre-treatment with the inhibitors (Figs. S4A and B). H&E staining revealed increased lung injury scores following anta-miR-744 treatment, which were alleviated by pre-treatment with the ACSL4 and ALOX15 inhibitors (Fig. 8D and E). ELISA revealed increased pulmonary MPO levels after anta-miR-744 treatment, which was mitigated by pre-treatment with the ACSL4 and ALOX15 inhibitors (Fig. 8F). Mouse lungs exposed to the GPX4 inhibitor exhibited increased ACSL4, ALOX15, IL-6, TNF-α, and MPO levels; augmented injury scores; and diminished GPX4 expression. However, miR-744 overexpression reversed these effects (Fig. 8C–F and S4A, B). These findings indicated that anta-miR-744 amplifies ferroptosis, inflammation, and injury in mouse lungs predominantly via regulation of GPX4, ACSL4, and ALOX15.

Following the procedure shown in Fig. 8G, we found that intratracheal administration of anta-miR-744 in BALB/c mice decreased pulmonary epithelial-cell miR-744 levels compared with those in the miR-NC group. Treatment with anta-miR-744 increased the epithelial-cell mRNA and protein levels of ACSL4 and ALOX15, decreased those of GPX4 (qPCR results in Fig. 8I and J; western blots in Fig. 8K), and increased the levels of ROS (Fig. S4C). These findings suggested that anta-miR-744 induced ferroptosis and inflammation in mouse epithelial cells.

### 3.10 miR-744 attenuates LPS-induced ferroptosis and inflammation in HBE cells and ARDS mice

**Fig. 9:**
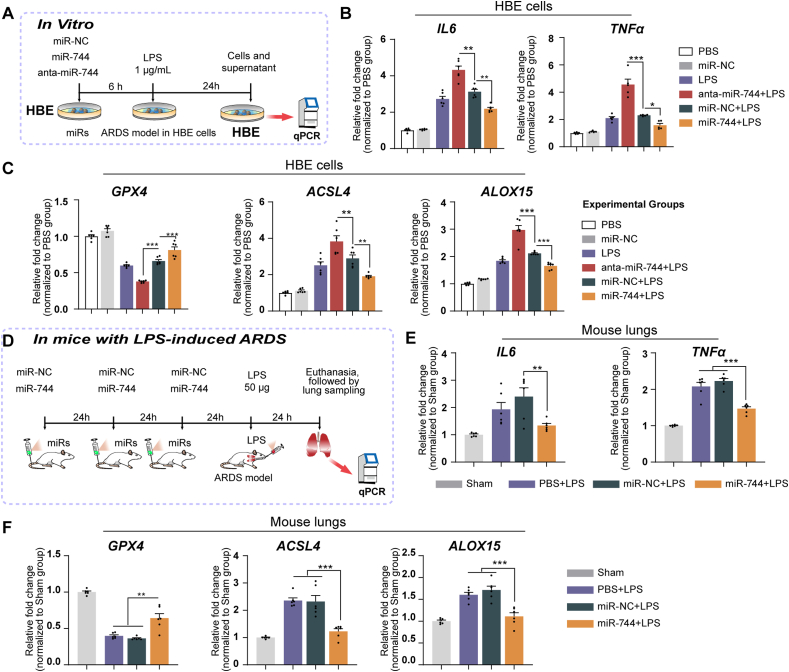
miR-744 attenuated LPS-induced ferroptosis and inflammation in HBE cells and mice. **(A)** Flowchart of cell assay. **(B**– **C)** HBE cells were transfected with miR-744 or anta-miR-744 and incubated with 1 μg/mL LPS. HBE cells were collected for qPCR analysis. The experiments were conducted with three independent technical replicates. **(B)** qPCR results for *IL6* and *TNFa* expression. **(C)** qPCR results for *GPX4*, *ACSL4*, and *ALOX15* expression. (**D)** Flowchart of experiment for investigating the role of miR-744 in LPS-induced ARDS. **(E**, **F)** BALB/c mice were administered miR-744 via tail vein injection, followed by intratracheal injection of 50 μL LPS (1 μg/μL) (*n* = 6 mice/group). **(E)** qPCR results for *IL6* and *TNFa* expression in mouse lungs. **(F)** qPCR results for *GPX4*, *ACSL4*, and *ALOX15* expression in mouse lungs. ∗*P* < 0.05, ∗∗*P* < 0.01, ∗∗∗*P* < 0.001 (ANOVA, followed by multiple comparison tests). *ACSL4*, long-chain acyl-CoA synthetase 4; *ALOX15*, 15-lipoxygenase; ARDS, acute respiratory distress syndrome; *GPX4*, glutathione peroxidase 4; HBE, human bronchial epithelial; LPS, lipopolysaccharide; miRNA, microRNA.

qPCR analysis revealed that in HBE cells pre-treated with miR-744 and exposed to LPS, *GPX4* mRNA expression increased while *ACSL4*, *ALOX15*, *IL6*, and *TNFa* expression decreased. This suggests that miR-744 exerts a protective effect against LPS-induced damage in these cells. In contrast, anta-miR-744 amplified the negative effects of LPS in HBE cells (Fig. 9A–C).

Pre-administration of miR-744 increased the expression of *GPX4* but decreased the expression of *ACSL4*, *ALOX15*, *IL6*, and *TNFa* mRNA in lung tissue of mice with LPS-induced ARDS (Fig. 9D–F). miR-744 pre-treatment resulted in analogous molecular patterns in mice with LPS-induced sepsis, further reinforcing the protective role of miR-744 against LPS-mediated damage in mouse lungs(Figs. S5A–C).

### 3.11 Expression of miR-744 was decreased in the lungs of mice with LPS-induced ARDS as well as plasma and exosomes of patients with ARDS

**Fig. 10:**
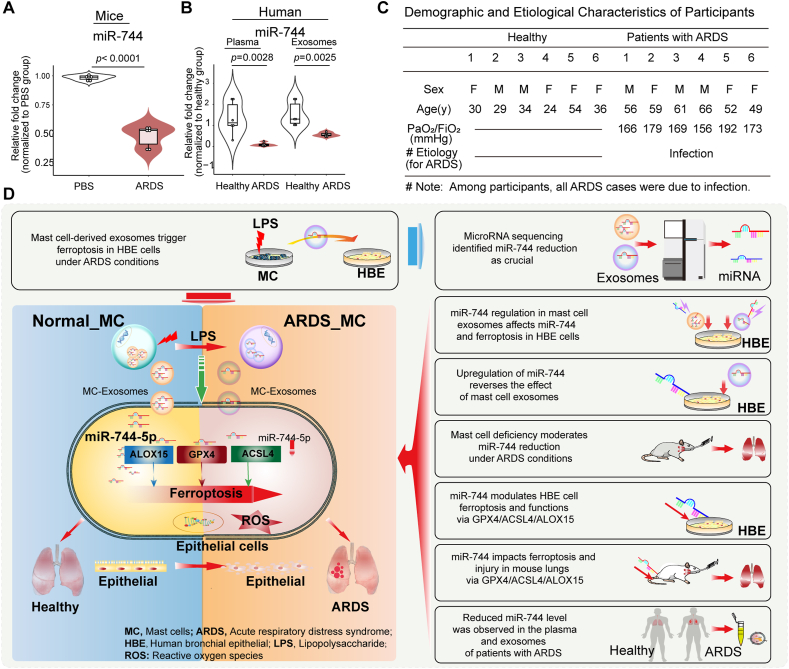
Pulmonary expression of miR-744 decreased in mice with LPS-induced ARDS and the plasma and exosomes of patients with ARDS. **(A)** qPCR results for miR-744 expression. BALB/c mice were intratracheally injected with 50 μL LPS (1 μg/μL) (*n* = 6 mice/group). **(B)** qPCR results for miR-744 expression in the plasma and exosomes of patients with ARDS. **(C)** Demographic and etiological characteristics of patients with ARDS. Unpaired Student's *t*-tests were used to evaluate the differences between groups. **(D)** Hypothetical mechanism by which miR-744 expression participates in ARDS development. *ACSL4*, long-chain acyl-CoA synthetase 4; *ALOX15*, 15-lipoxygenase; ARDS, acute respiratory distress syndrome; *GPX4*, glutathione peroxidase 4; HBE, human bronchial epithelial; LPS, lipopolysaccharide; miRNA, microRNA.

The expression of miR-744 was markedly reduced in the LPS-induced ARDS mouse model (Fig. 10A). Compared with healthy volunteers, we observed a marked decline in miR-744 expression in the plasma and exosomes of patients with ARDS (Fig. 10B). The demographic and etiological characteristics of participants in this study are shown in Fig. 10C. A schematic representation of the hypothesized mechanism and study design is provided in Fig. 10D.

## 4 Discussion

This study elucidated a key mechanism by which mast cell-derived exosomes mediate epithelial cell ferroptosis through reduced miR-744 expression, offering novel insights into the pathogenesis of ARDS. Our findings highlight the crucial role of miR-744 in ARDS and its potential for diagnostic and therapeutic applications. The downregulation of miR-744 expression in mast cell-derived exosomes was a critical factor for inducing ferroptosis and inflammation in HBE cells under ARDS conditions via the regulation of miR-744, GPX4, ALOX15, ACSL4, IL-6, and TNF-α levels, thereby influencing cell viability and migration through a novel pathogenic mechanism. The upregulation of miR-744 in HBE cells reversed these effects, while its downregulation in anta-miR-744-transfected cells further exacerbated ferroptosis and inflammation. In BALB/c mice with LPS-induced ARDS, this downregulation promoted pulmonary ferroptosis, inflammation, and injury, underscoring the pivotal role of miR-744 in epithelial cell injury and ARDS development *in vivo*. These effects were modulated by the activities of ALOX15, ACSL4, and GPX4, as evidenced by the effects of their specific inhibitors. The therapeutic potential of miR-744 was supported by its effectiveness in mitigating pathological changes in LPS-challenged HBE cells and BALB/c mice. Additionally, reduced miR-744 levels in the plasma and exosomes of patients with ARDS indicated its diagnostic value.

LPS, a major pathogenic factor in ARDS, is commonly used to establish classical ARDS models [22,29,30]. HMC-1 cells, which exhibit properties similar to lung mast cells, were used in this study to mimic mast cell exosome production under ARDS conditions [8,31]. Our findings demonstrated that mast cell-derived exosomes influence ferroptosis and inflammation in epithelial cells, and LPS exposure amplified these effects. This suggests that mast cell-derived exosomes enhance epithelial cell susceptibility to LPS, consistent with the “multi-hit” nature of ARDS [25], and may exacerbate inflammation [32], highlighting the crucial role of mast cell–epithelial interactions in ARDS development.

Consistent with the findings of previous studies [11], LPS treatment resulted in significant alterations in the miRNA profiles of mast cell-derived exosomes, contributing to the involvement of mast cells in various diseases [33,34]. The reduction in mast cell-derived exosomal miR-744 expression, along with results from mast cell-deficient mice, suggests a new role for mast cells in maintaining epithelial balance under normal conditions, where miR-744 has a protective effect [35]; however, this mechanism may exacerbate ARDS. Mast cells contribute to ARDS through degranulation and mediator production [5,36]. Given the long-lasting effects of LPS on mast cells [22] and their inherent longevity [37], this mast cell-derived mechanism may sustain ARDS development beyond the immediate immune response long after pathogen clearance [5,[38], [39], [40]]. Our findings reveal a novel role for mast cells in ARDS that warrants further exploration.

Consistent with the findings of previous studies [41,42], we found that the downregulation of mast cell-derived exosomal miR-744 expression reduced its expression in HBE cells, potentially inhibiting cellular productivity [41,42]. miRNAs regulate gene expression by binding to specific mRNAs [10,43]. Our study indicated a significant association between miR-744 and key ferroptosis-related genes, particularly *GPX4*, *ACSL4*, and *ALOX15*. Ferroptosis-related epithelial cell injury is a key driver of LPS-induced ARDS [44,45]. Mechanistically, the downregulation of miR-744 leads to mitochondrial damage and cytosolic iron release [46]. This released iron participates in the Fenton reaction, producing ROS that drive lipid peroxidation and ferroptosis, leading to cell death [[47], [48], [49]]. ROS feedback loops, including the Fenton reaction and Nicotinamide adenine dinucleotide phosphate oxidase signaling, further amplify ferroptosis and inflammation [50]. Our study underscores the critical regulatory role of miR-744: in normal tissues, miR-744 suppresses ferroptosis to maintain cellular functions, whereas its reduced expression in ARDS induces ferroptosis, leading to inflammation and cellular dysfunction. Targeting ferroptosis to mitigate inflammation and injury presents a novel therapeutic strategy for ARDS.

Our investigation of clinical samples emphasize the therapeutic potential of miR-744 in ARDS, though further validation is required. miRNAs are important regulators of various pathological conditions, and their therapeutic potential is an area of growing research [51,52]. Previous studies on ARDS have attempted to maintain their pulmonary levels via pre-ARDS miRNA administration [23]. miR-744 has also been identified as a potential biomarker in diseases such as alcoholic hepatitis [53] and heart failure [54]. Our findings suggest that miR-744 could serve as a diagnostic and/or therapeutic target in ARDS, although large-scale preclinical studies are needed to confirm its utility before clinical trials can be conducted.

This study has some limitations. First, the mechanisms driving LPS-induced miRNA alterations in mast cell exosomes warrant further investigation. Second, the introduction of miR-744 into the mouse lungs may have triggered ferroptosis in other cell types. Future studies should investigate the effects of miRNAs on diverse lung tissue cells. Nevertheless, our findings highlight the critical role of mast cell-derived exosomal miR-744 in ferroptosis and the pathogenesis of ARDS in LPS-challenged HBE cells. These insights can inform the development of therapeutic strategies targeting mast cells and miR-744-induced ferroptosis to prevent and/or treat ARDS. Further preclinical and clinical translational research should focus on the therapeutic and diagnostic value of miR-744.

## 5 Conclusions

This study demonstrated that mast cell-derived exosomes induce ferroptosis and inflammation in epithelial cells under LPS-induced ARDS, with reduced exosomal miR-744 levels identified as a crucial factor. The regulation of miR-744 levels in mast cell-derived exosomes altered miR-744 expression, ferroptosis, and inflammation in epithelial cells. miR-744 regulated the GPX4/ACSL4/ALOX15 axis to induce ferroptosis, inflammation, and injury in both cellular and animal models of LPS-induced ARDS. These novel findings underscore the critical role of miR-744 in epithelial cell injury and ARDS development. The restoration of miR-744 expression attenuated LPS-induced pathological changes *in vivo*, highlighting its potential as a therapeutic agent. Furthermore, the reduction of miR-744 levels in patients with ARDS highlight its diagnostic potential in ARDS. Overall, our findings provide valuable insights into ARDS pathogenesis and provide a useful reference for treatment development. Future translational studies should focus on the diagnostic and therapeutic potential of miR-744 in clinical settings.

## Patient consent statement

Patient consent was obtained prior to participation, in accordance with ethical standards.

## Funding

This work was supported by the Joint Funds for the Innovation of Science and Technology of Fujian Province [grant number 2023Y9339]; Fujian Provincial Health Technology Project [grant number 2023CXA006]; Fujian Medical University Startup Fund for scientific research [grant number 2021QH1320]; National Natural Science Foundation of China[grant number 82171186 and 82471278)]; and Joint Funds for the Innovation of Science and Technology of Fujian Province [grant number 2023Y9282].

## CRediT authorship contribution statement

**Xiaobin Fang:** Writing – review & editing, Writing – original draft, Supervision, Resources, Project administration, Methodology, Funding acquisition, Formal analysis, Conceptualization. **Fei Gao:** Visualization, Resources, Project administration, Methodology, Investigation, Formal analysis, Data curation. **Ling Zheng:** Visualization, Investigation, Funding acquisition. **Fu-Shan Xue:** Writing – review & editing, Supervision. **Tao Zhu:** Writing – review & editing, Supervision, Project administration. **Xiaochun Zheng:** Writing – review & editing, Supervision, Project administration, Funding acquisition.

## Declaration of Generative AI and AI-assisted technologies in the writing process

None.

## Declaration of competing interest

The authors declare that they have no known competing financial interests or personal relationships that could have appeared to influence the work reported in this paper.

## Data Availability

Data will be made available on request.
