# Supplementary material for: Reduced microRNA-744 expression in mast cell-derived exosomes triggers epithelial cell ferroptosis in acute respiratory distress syndrome
Source: Redox Biol. 2024 Oct 3;77:103387. doi: 10.1016/j.redox.2024.103387 (PMC11493202; doi:10.1016/j.redox.2024.103387)
Supplement: Multimedia component 1 [file mmc1.docx]

Supplementary Material

**Reduced microRNA-744 expression in mast cell-derived exosomes triggers epithelial cell ferroptosis in acute respiratory distress syndrome**

Xiaobin Fang^a,^*^,‡^, Fei Gao^a,‡^, Ling Zheng^a^, Fu-Shan Xue^a^, Tao Zhu^b,^*, and Xiaochun Zheng^c,^*

^a^ Department of Anesthesiology/Critical Care Medicine, Shengli Clinical Medical College of Fujian Medical University, Fujian Provincial Key Laboratory of Critical Care Medicine, Fujian Provincial Hospital, Fuzhou University Affiliated Provincial Hospital, Fuzhou, Fujian 350001, China.

^b^Department of Anesthesiology, West China Hospital, Sichuan University & The Research Unit of West China (2018RU012), Chinese Academy of Medical Science, Chengdu, Sichuan, China

^c^Department of Anesthesiology, Fujian Provincial Hospital, Shengli Clinical Medical College of Fujian Medical University & Fujian Emergency Medical Center, Fujian Provincial Key Laboratory of Emergency Medicine, Fujian Provincial Key Laboratory of Critical Medicine, Fujian Provincial Co-constructed Laboratory of “Belt and Road,” Fuzhou, Fujian, China

**Supplementary figures**

**
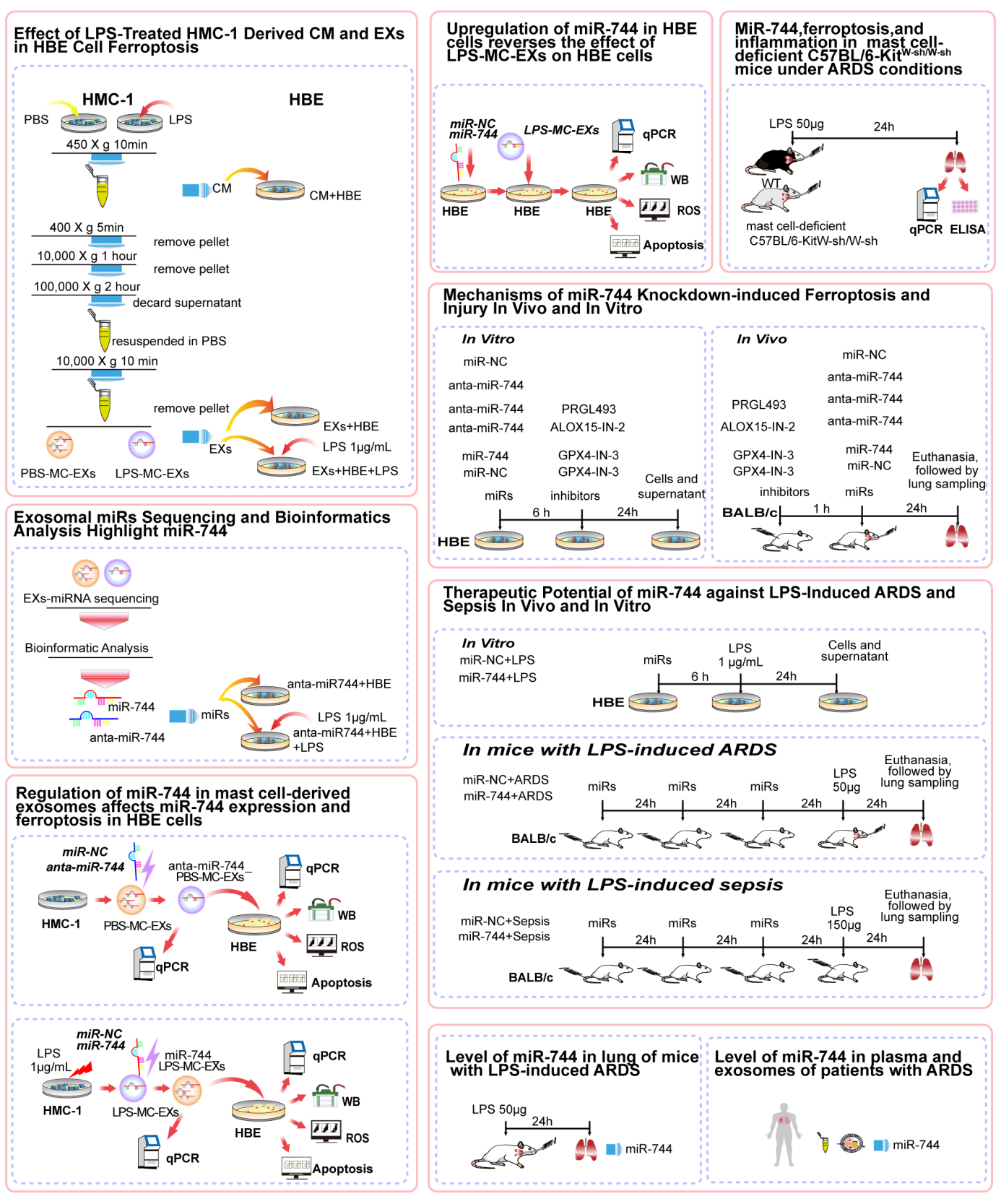
**

**Fig.S1. Flowchart of this study.**

EXs: exosomes; CM: conditioned medium; LPS: lipopolysaccharide; HBE: bronchial epithelial cells; HMC-1: human mast cell line 1. ACSL4, long-chain acyl-CoA synthetase 4; ALOX15, 15-lipoxygenase; ARDS, acute respiratory distress syndrome; GPX4, glutathione peroxidase 4; PBS, phosphate-buffered saline; LPS-MC-CM, conditioned medium from HMC-1 cells stimulated with LPS; PBS-MC-CM, conditioned medium from HMC-1 cells stimulated with PBS; LPS-MC-EXs, exosomes from HMC-1 cells stimulated with LPS; PBS-MC-EXs, exosomes from HMC-1 cells stimulated with PBS.


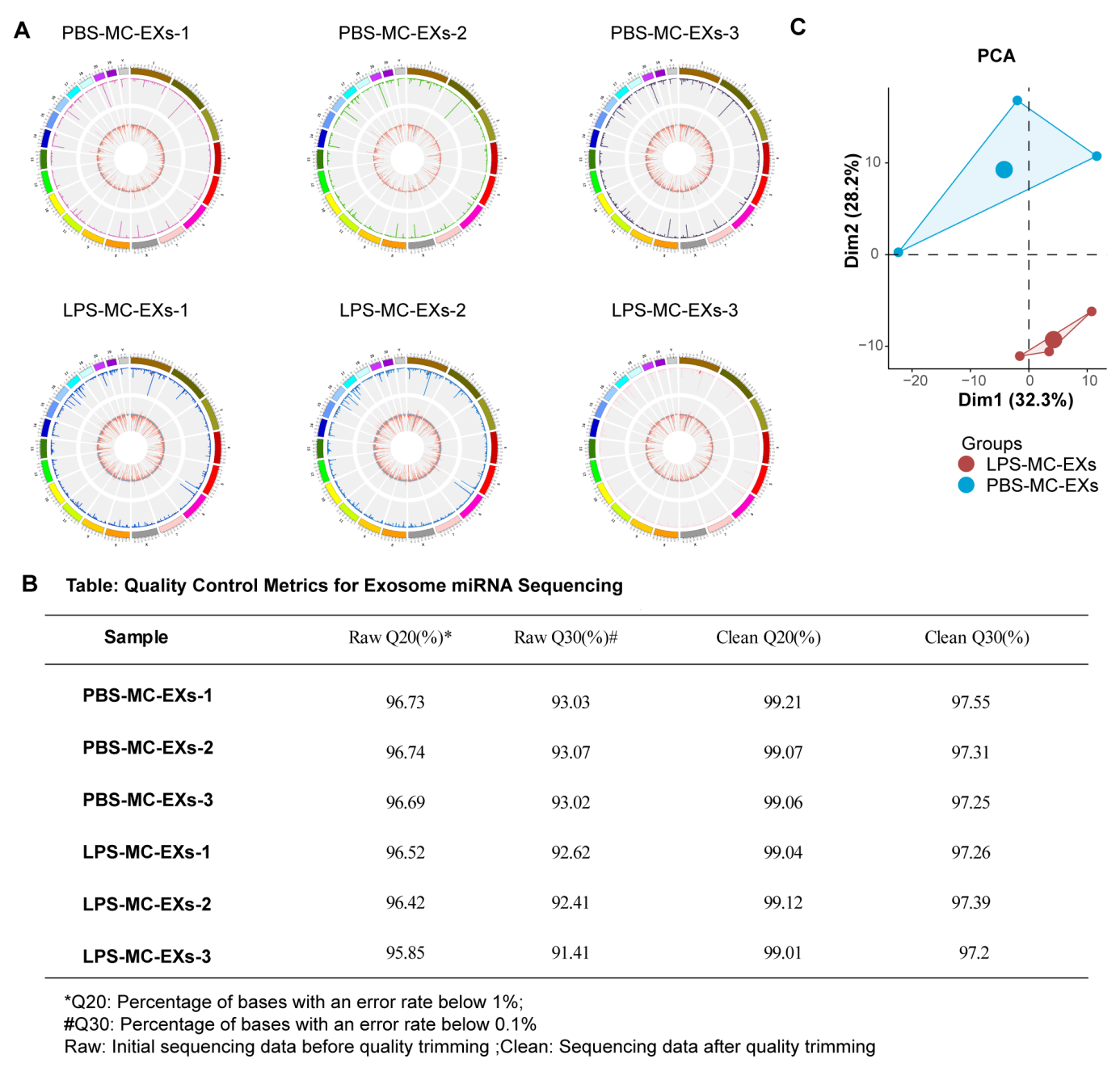


**Fig.S2. Exosomal miRNA Screening. (A)** Circos plots for exosomes-miRNA sequence. **(B)** sequencing quality. **(C)** PCA results. PBS-MC-CM, conditioned medium from HMC-1 cells stimulated with PBS; EXs, exosomes; LPS-MC-EXs, exosomes from HMC-1 cells stimulated with LPS; PBS-MC-EXs, exosomes from HMC-1 cells stimulated with PBS.


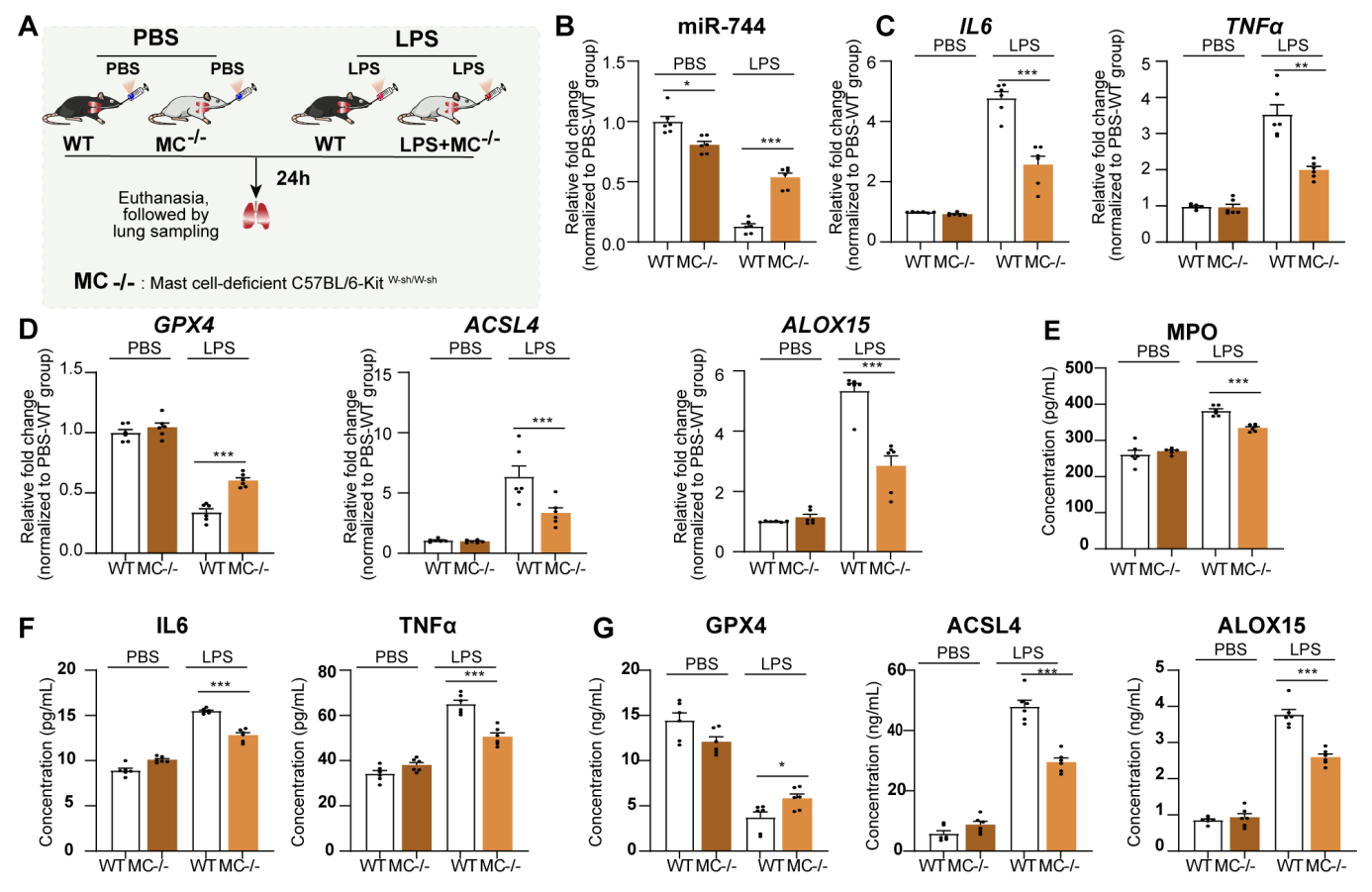


**Fig.S3. Mast cell-deficient mice exhibit slightly reduced miR-744 expression and milder pulmonary inflammation and ferroptosis under ARDS conditions.** **(A)** Flowchart of the experiment for inducing the ARDS model using LPS in mast cell-deficient mice. **(B)** qPCR analysis results for miR-744. **(C)** qPCR analysis results for *IL6* and *TNFα*. (**D)** qPCR analysis results for *GPX4*, *ACSL4*, and *ALOX15*. **(E)** ELISA results for myeloperoxidase activity. **(F)** ELISA results for *IL6* and *TNFα*. **(G)** ELISA results for *GPX4*, *ACSL4*, and *ALOX15*. ANOVA, followed by multiple comparison tests, was used to evaluate the differences between groups (*, *P* < 0.05; **, *P* < 0.01; ***, *P* < 0.001). *ACSL4*, long-chain acyl-CoA synthetase 4; *ALOX15*, 15-lipoxygenase; *GPX4*, glutathione peroxidase 4; HBE, human bronchial epithelial; LPS, lipopolysaccharide; miRNA, microRNA; qPCR, quantitative real-time PCR.


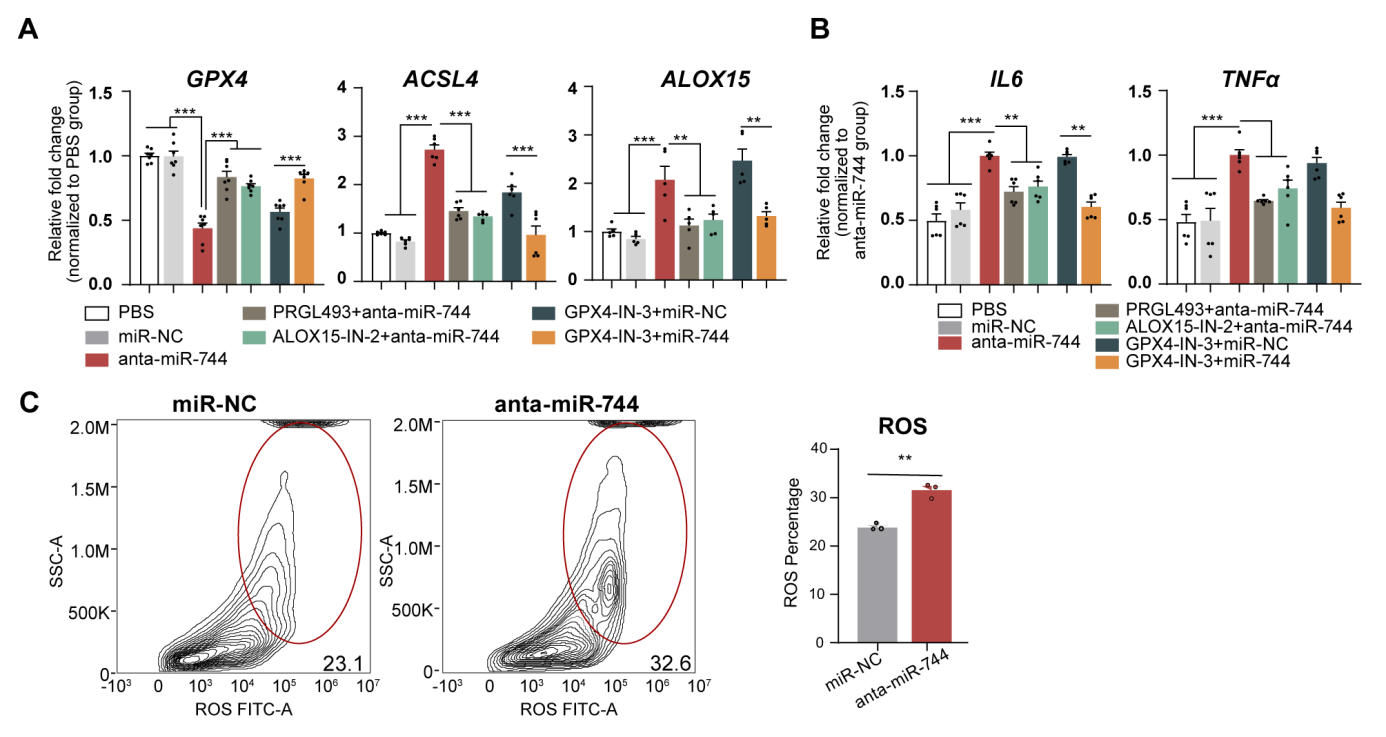


**Fig.S4. Intratracheal administration of anta-miR-744 modulates ferroptosis, inflammation, and injury in mouse lungs via the expression of GPX4, ACSL4, and ALOX15. (A)- (B)** BALB/c mice were administered anta-miR-744 intratracheally, and lungs were collected for analysis after 24 hours. N=6 per group. ANOVA, followed by multiple comparisons, was used to evaluate the differences between groups; **(A)** qPCR analysis results for *GPX4*, *ACSL4*, and *ALOX15*. **(B)** qPCR analysis results for *IL6* and *TNFα*. **(C)** Epithelial cells were isolated from the mice lungs for analysis. Student's t-test was conducted to evaluate the differences between groups (*, *P* < 0.05; **, *P* < 0.01; ***, *P* < 0.001). **(C)** Flow cytometry data for ROS, including representative images (Left) and quantitative analysis (Right). *ACSL4*, long-chain acyl-CoA synthetase 4; *ALOX15*, 15-lipoxygenase; *GPX4*, glutathione peroxidase 4; HBE, human bronchial epithelial; H&E, hematoxylin and eosin; miRNA, microRNA


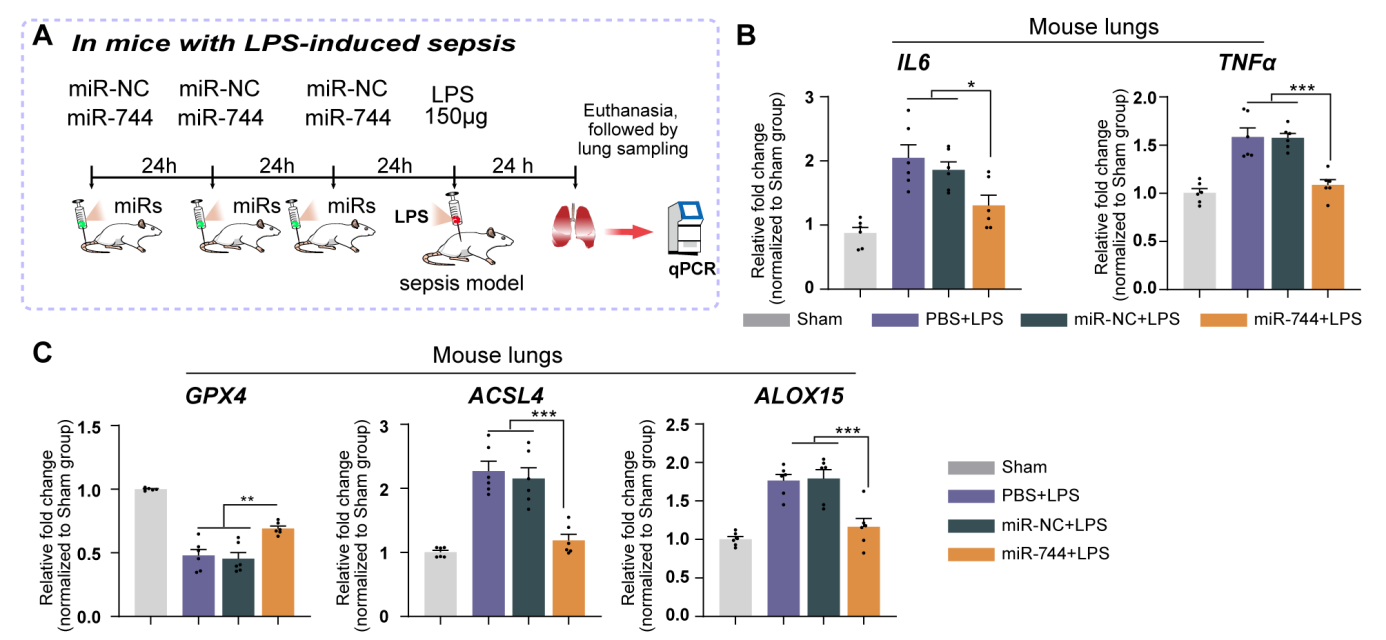


**Fig.**S5. miR-744 attenuates LPS-induced ferroptosis and inflammation in mice. BALB/c mice were administered miR-744 via tail vein injection, followed by intraperitoneal injection of 50 μL(3 μg/μL) LPS. Lungs were collected for analysis. N=6 per group. **(A)** Flowchart of the cell assay. **(B)**qPCR analysis results for *IL6* and *TNFα*. **(C)** qPCR analysis results for *GPX4*, *ACSL4*, and *ALOX15*. ANOVA, followed by multiple comparison tests, was used to evaluate the differences among groups (*, *P* < 0.05; ***, *P* < 0.001). *ACSL4*, long-chain acyl-CoA synthetase 4; *ALOX15*, 15-lipoxygenase; ARDS, acute respiratory distress syndrome; *GPX4*, glutathione peroxidase 4; HBE, human bronchial epithelial; LPS, lipopolysaccharide; miRNA, microRNA.

**Supplementary tables**

**Table S1: Information on the reagents in current study.**

| **Materials** | **Species** | **Manufacturer** | **Vendor details** |
| --- | --- | --- | --- |
| anit-ALIX antibody | Human | Abcam (Cambridge, UK) | Cat: ab76608 |
| anit-CD81 antibody | Human | Abcam (Cambridge, UK) | Cat: ab219209 |
| anit-CD31 antibody | Human /Mouse | Abcam (Cambridge, UK) | Cat: ab9498 |
| anit-tubulin antibody | Human /Mouse | Proteintech (Wuhan, China) | Cat:.66031-1-lg |
| anti-GPX4 antibody | Human /Mouse | Abcam (St. Louis, MO, USA) | Cat: ab223582 |
| anti-ACSL4 antibody | Human /Mouse | Affinity (Jiangsu, China) | Cat:DF12141 |
| anti-CD45 antibody | Mouse | BioLegend (San Diego, CA, USA). | cat:147711 |
| anti-CD326 antibody | Mouse | BioLegend (San Diego, CA, USA). | cat:118213 |
| anti-ALOX15 antibody | Human /Mouse | Abcam (St. Louis, MO, USA) | Cat: ab244205 |
| RPMI 1640 |  | GLPBIO (Shanghai, China) | Cat:8123259 |
| ELISA kits for myeloperoxidase | Mouse | Mbbiology (Jiang su, China) | Cat: MB-3074A |
| LPS *(Escherichia coli)* |  | Solarlio Life Sciences (Beijing, China) | L8880 |
| GPX4-IN-3 |  | GLPBIO (Shanghai, China) | Cas No.2761004-85-5 |
| PRGL493 |  | GLPBIO (Shanghai, China) | Cas No.2479378-45-3 |
| ALOX15-IN-2 |  | GLPBIO (Shanghai, China) | Cas No.2764818-23-5 |
| Dil [dilc 18(3)] |  | MCE (New Jersey，USA) | Cat: hy-d0083 |
| Calcein-AM (Calcein acetoxymethyl ester) |  | GLPBIO (Shanghai, China) | Cas No.:148504-34-1 |
| Deferiprone |  | GLPBIO (Shanghai, China) | Cas No.:30652-11-0 |
| FITC Annexin V | Human /Mouse | BioLegend, San Diego, CA, USA | Cat#640905 |
| ROS Assay Kit | Human /Mouse | Beyotime(Shanghai,China) | Cas:S0033S |
| ELISA kits for GPX4 | Mouse | Ruixinbio(Fujian, China) | Cat.# RX200682M |
| ELISA kits for ACSL4 | Mouse | Ruixinbio(Fujian, China) | Cat,# RX201364M |
| ELISA kits for ALOX15 | Mouse | Ruixinbio(Fujian, China) | Cat# RX203810M |
| ELISA kits for IL6 | Mouse | Elabscience(Wu han,China) | Cat: E-EL-M0044c |
| ELISA kits for TNFα | Mouse | Elabscience(Wu han,China) | Cat:E-MSEL-M0002 |
| lung dissociation kit |  | Miltenyi Biotec(Bergisch Gladbach, Germany) | Cat. No. 130-095-927) |

**Table S2. Sequences of miRNAs used in Experiments.**

| **primers** | **Sequences** |
| --- | --- |
| #miR-744 | UGCGGGGCUAGGGCUAACAGCA |
| #Anta-miR-744 | UGCUGUUAGCCCUAGCCCCGCA |

#Note: The listed miRNA sequences are identical in both human and mouse.

**Table S3****. The primers used for qPCR in our study.**

| **primers** | **Forward** | **Reverse** |
| --- | --- | --- |
| GPX4-Human | CCGCTGTGGAAGTGGATGAAGATC | CTTGTCGATGAGGAACTGTGGAGAG |
| ACSL4-Human | CTCCAAGTAGACCAACGCCTTCAG | GGTCCCAGTCCAGGTATTCTTTCAC |
| ALOX15-Human | ACCTTCCTGCTCGCCTAGTGTTC | GTGCTGCTGGCTACAGAGAATGAC |
| GPX4-Mouse | CCGAGTTCCTGGGCTTGTGTG | CCGTCGATGTCCTTGGCTGAG |
| ACSL4-Mouse | CACCCACTCCCATCTCCCGAAG | TGGCATCTCCCTGGTCCCTTAAC |
| ALOX15-Mouse | CGCAGCACTCTTCCATCCATCTTG | CGTCGCCATCAGCTTCTCCATC |
| IL6-Human | CAATATTAGAGTCTCAACCCCCAA | TCACCAGGCAAGTCTCCTCA |
| IL6-Mouse | GGAGCCCACCAAGAACGATAGTC | CACCAGCATCAGTCCCAAGAAGG |
| TNFα-Human | CTCGAACCCCGAGTGACAAG | TGAGGTACAGGCCCTCTGAT |
| TNFα-Mouse | GGACTAGCCAGGAGGGAGAACAG | GCCAGTGAGTGAAAGGGACAGAAC |

.

**Supplementary texts**

**1. Materials**

MiRNAs were purchased from Synbio technologies (SuZhou,China; Project number: T34599). Other reagents were of analytical grade and were obtained from Sigma-Aldrich (St. Louis, MO, USA). **Table S1** enumerates the reagents employed in this study.

**2: Detailed cell assays procedures**

**Cell culture, exosome isolation, and characterization**

HMC-1 and HBE135-E6E7 cells (HBE) were obtained from the Shanghai Cell Bank of the Chinese Academy of Sciences (Shanghai, China), and cultured in RPMI 1640 (Cat:8123259, Gibco) supplemented with 100 μg/mL streptomycin, 100 U/mL penicillin, and 10% heat-inactivated fetal bovine serum (FBS). Cells were maintained at 37°C in a humidified atmosphere containing 5% CO_2_.

Fresh culture medium was subjected to ultracentrifugation at 100,000 g for 2 hours to deplete exosomes before used. HMC-1 cells, at a density of 1,000,000 cells/mL, were washed twice with PBS. They were then resuspended in 1 mL of the exosome-depleted culture medium. Depending on the treatment group, cells were either exposed to 10 μL of PBS or LPS (100 ng/μL). After a 24-hour incubation, both cells and their supernatant were collected.

The collected supernatant underwent sequential centrifugation: first at 400×g for 5 minutes, then at 10,000×g for 1 hour, and finally at 100,000×g for 2 hours to isolate exosomes. The resulting exosome pellet was washed with PBS, resuspended in 1 mL of PBS, centrifuged at 10,000×g for 10 minutes, and subsequently filtered through a 0.22 μm filter for storage and subsequent experiments. The protein content of the exosomes was determined using the Pierce™ BCA Protein Assay kit (Thermo Fisher Scientific Inc). All exosomes were diluted to a concentration of 1 µg protein/µL using PBS. Exosome characterization was conducted using transmission electron microscopy (TEM), nanoparticle tracking analysis (NTA), and western blotting.

For TEM analysis, 10 μL of the sample was placed on a grid and negatively stained with 3% uranyl acetate for 1 minute. Transmission images were captured using an HT-7700 TEM (Hitachi, Tokyo, Japan). For NTA, 50 μL of exosomes was diluted in 50 mL of PBS and analyzed using a ZETA VIEW instrument (Particle Metrix, Munich, Germany) and the associated Zeta View Electrophoresis and Brownian Motion Video Analysis software. For western blotting, exosome samples were lysed in 100 μL of RIPA buffer and subjected to sodium dodecyl sulfate-polyacrylamide gel electrophoresis on 12% gels. Primary antibodies used included anti-ALIX and anti-CD81.

**Exosome labeling with DiI and uptake study**

To evaluate the *in vitro* uptake of exosomes by HBE cells, the purified exosomes were labeled with Dil using Dilc18(3) kit (New Jersey, USA) following the manufacturer's instructions. The Dil concentration was adjusted to 10 µM per microliter of exosome solution. The exosomes were stained with Dil dye in 1 mL of the exosome solution for 15 minutes at room temperature. After staining, the labeled exosomes were washed to remove unbound dye and then incubated with HBE cells for 24 hours at 37°C. Images were captured using an Olympus automated inverted fluorescence microscope (Olympus Corporation, Tokyo, Japan).

**Co-culture stimulation of HBE cells with HMC-1 supernatant and exosomes**

Prepared HBE cells were treated in 1 mL of exosome-depleted medium. The treatments were divided into six groups: 50 μL of HMC-1 conditioned medium (CM), either stimulated with PBS (PBS-MC-CM) or LPS (LPS-MC-CM); 50 μL of exosomes from HMC-1, post-stimulation with PBS (PBS-MC-EXs) or LPS (LPS-MC-EXs); 50 μL of either PBS-MC-EXs or LPS-MC-EXs, each followed by a 10 μL of LPS (100 ng/μL) treatment. After 24 hours, cells and supernatant from each group were collected.

**MicroRNA sequencing of exosomes**

Exosomal miRNA sequencing was conducted with the help of Kangce Technology Co., Ltd. (Wuhan, China, Project No: KC2023-H0149). RNAs were extracted from EXs using TRIzol Reagent (InVitrogen, USA) and assessed for quality and integrity via the Bioptic Qsep100. Post-quantification, stranded RNA sequencing libraries were prepared. Sequencing data was mapped to the human genome via STRA 2.5 software. Differentially expressed miRNAs (DE_miR) were analyzed with EdgeR package of R software (4.2.3). All raw sequencing data have been submitted to the Gene Expression Omnibus (GEO) database under accession number GSE (<https://www.ncbi.nlm.nih.gov/geo/>).

**Bioinformatic analysis of differentially expressed miRNAs**

Data were processed and analyzed using R software. Principal Component Analysis (PCA) evaluated inter-group correlations. Initial screening was conducted using was conducting using volcano plots with a significance threshold of *P*-value < 0.1 and |log(fold change)| > 1.5. DE_miRs between LPS-MC-EXs and PBS-MC-EXs were visualized using heatmaps. MultiR package, encompassing data from 14 databases, predicted regulatory miRNAs for ACSL4/ALOX15/GPX4. The intersection of three predicted miRNA groups (regulating ACSL4, ALOX15, and GPX4) with De_miRs was performed to identify the target miRNAs. Regulated genes of the target miRNAs were predicted using the multiR package. Ferroptosis-associated genes were sourced from the ferroptosis website (<http://www.zhounan.org/ferrdb/current/>). Regulated genes of target miRNAs were intersected with these genes to show the relationship between the target miRNAs and ferroptosis. Identified miRNAs were transfected into HBE cells using mimics for upregulated and antagonists for downregulated miRNA.

**Transfection of exosomes with miRNA mimics or antagonists**

Exosomes were isolated following the protocol in “***Cell Culture, Exosome Isolation, and Characterization***” of Methot section. Electroporation of exosomes was conducted using the NEPA21 electroporator (NEPA GENE Co., Ltd., Chiba, Japan). A 100 μL exosome suspension mixed with 1.5 μL of 20 μg/mL miRNA solution was transferred to a 2 mm gap electroporation cuvette. The electroporation parameters were set to 100 V, 5 ms pulse width, and 2 pulses. The electrical resistance was kept between 30 Ω and 50 Ω. After electroporation, the samples were collected and stored for future use.

**Transfection of HBE cells with miRNA mimics or antagonists**

For miRNA transfection experiments, HBE cells were seeded into six-well plates at a concentration of 10^6^ cells/mL. Transfections were performed using lipofectamine 3000 reagent according to the manufacturer's protocol. Briefly, lipofectamine 3000 Reagent (5 μL) was diluted in 250 μL RPMI 1640 reduced serum medium, and miRNA solutions (5 μL, 120 μg/mL) were prepared in a separate 250 μL aliquot of RPMI 1640 reduced serum medium. The two solutions were combined and incubated for 10 minutes before being introduced to the cells. After a 6-hour incubation, each well received 1 mL of RPMI 1640 medium, and inhibitors for ACSL4, ALOX15, and GPX4 were added as required. Cells and supernatants were collected 36 hours post-transfection for further analyses.

HMC-1 cells were transfected with a variety of miRNAs, including miR-NC (negative control), miR-744, anta-miR-744. The sequences of these miRNAs are provided in **Table S2**.

To investigated the response of HBE after miR-744 transfection, HBE was transfected by miR-NC, miR-744 or anta-miR-744. Cells and supernatants were collected for analysis. To test the modulation of ACSL4, ALOX15, and GPX4, HBE that have been transfected with miR-744 or anta-miR-744 were incubated with PRGL493(a ACSL4 inhibitor) at 5 µM, ALOX15-IN-2 (a ALOX15 inhibitor) at 5 µM, and GPX4-IN-3(a GPX4 inhibitor) at 2 µM, cells were collected for further analyses.

**Dual-luciferase reporter assay**

Wild-type and mutant plasmids for GPX4, ACSL4, and ALOX15 were provided by GentleGen Biotech (Suzhou, China; Contract Number: G245196). The plasmids were transfected into HBE cells using Lip3000 for 24 hours, followed by the introduction of either miR-NC or miR-744. The assay was then conducted in accordance with the manufacturer's protocol.

**TEM for HBE cells**

After transfection with anta-miR-744 and miR-NC, HBE cells were prepared for electron microscopy as follows: Prefixed in 3% glutaraldehyde, postfixed in 1% osmium tetroxide, dehydrated in acetone, infiltrated and embedded in Epon 812. Sections were stained with methylene blue for semithin cuts and with uranyl acetate and lead citrate for ultrathin cuts, then examined using a JEM-1400-FLASH Transmission Electron Microscope.

**Labile iron pool Assay**

Labile iron pool (LIP) levels were assessed utilizing calcein-acetoxymethyl ester (calcein-AM, MedChemExpress, NJ, USA) and deferiprone (MedChemExpress), in accordance with the methodologies delineated in the literature. HBE cells were seeded onto 6-well plates at a density of 1 × 10^6^ cells per well. Following treatment, cells were incubated with 0.5 μM Calcein AM for 15 minutes at 37°C. Subsequently, cells were incubated with deferiprone for 1 hour at 37°C. The medium containing deferiprone was then replaced with fresh medium, and cells were examined and imaged using a fluorescence microscope. LIP Assay was used to determine free iron levels.

**CCK8 assay**

HBE cells (1×10^6 cells/mL) were seeded in 96-well plates and subjected to various treatments. Cell viability was measured at 0 hours, 24 hours, 48 hours, and 72 hours post-treatment. Specifically, at each time point, 10 µL of CCK8 solution (Dojindo, Japan) was added to each well, and the cells were further incubated for 90 minutes at 37°C with 5% CO2. The optical density (OD) at 450 nm was then measured using a spectrophotometer. The relative cell viability was calculated as the ratio of the OD value of the treatment groups to that of the PBS group.

**Trans-well invasion assay**

For the Trans-well invasion assay with HBE cells, cells (1×10^6 cells/mL) were seeded into the upper chamber of a Trans-well insert precoated with Matrigel. The lower chamber was filled with medium containing 10% FBS as a chemoattractant. Cells were incubated at 37°C with 5% CO2 for 24 hours to allow migration through the Matrigel and the porous membrane to the lower chamber. At the end of the experiment, cells in the upper chamber were fixed with 4% formaldehyde and stained with 0.1% crystal violet for 30 minutes. Non-migratory cells on the upper side of the membrane were gently removed with a cotton swab. Migrated cells on the lower side of the membrane were counted under a microscope.

**Scratch assay**

HBE cells (1×10^6 cells/mL) were seeded in 6-well plates and subjected to various treatments. Once cells reached 90% confluence, a straight scratch was made in the cell monolayer using a 200µL pipette tip. After scratching, cells were washed twice with serum-free medium to remove floating cells and debris, followed by the addition of fresh medium. Images of the scratch were captured at 0, 24, 48, and 72 hours using an inverted microscope.

**Flow cytometer**

HBE cells (1×10^6 cells/mL) were seeded in 6-well plates and subjected to various treatments. Cells were collected at 24 hours post-treatment for subsequent apoptosis analysis. Specifically, after collection, cells were washed with phosphate-buffered saline (PBS) and resuspended in binding buffer. Following the instructions of the Annexin V-FITC/PI double staining kit, Annexin V-FITC and PI were added to the cell suspension and incubated in the dark for 15-20 minutes. Post-incubation, fluorescence intensities in the FITC and PI channels were measured using a flow cytometer to distinguish between live cells, early apoptotic cells, and late apoptotic or necrotic cells. The percentages of each cell subpopulation were calculated using FlowJo software to assess the rate of cell apoptosis.

**3: Detailed animal experiment procedures**

**Animals**

Male BALB/c mice (20-22 g, 8-12 weeks) were obtained from DOSSY (Chengdu, China). Male mast cell-deficient C57BL/6-Kit^W-sh/W-sh^ and the corresponding C57BL/6-Kit^+/+^ wild-type mice (20-22 g, 8-12 weeks) were purchased from Cyagen (Jiangsu, China, c001378). All mice were housed in the animal room at the Experimental Animal Center of Fujian Provincial Hospital. Mice were fasted for 8 hours before the experiment, with free access to water. Groups of six mice were formed using R software, with an additional 10% included to account for potential losses. Any deceased mouse was replaced in a predetermined sequence.

**Intratracheal injection and treatment protocols**

Mice were anesthetized using 1% sodium pentobarbital (50 mg/kg) and underwent endotracheal intubation with arterial catheterization. Each mouse received a 50 µl intratracheal injection of either miR-NC, miR-744, or anta-miR-744 (2 µmol/µL), followed by 5 minutes of ventilation. Post-injection, mice were placed on a warm pad for recovery. After 24 hours, mice were euthanized with an overdose of sodium pentobarbital (100 mg/kg, intraperitoneally) for sample collection.

**Part 1: Investigating the effect of anta-miR-744 on lung function and the modulation of ACSL4, ALOX15, and GPX4.**

miR-NC Group: 50 µl miR-NC intratracheal injection.

Anta-miR-744 Group: 50 µl anta-miR-744 intratracheal injection.

ACSL4 Inhibitor + Anta-miR-744 Group: Intraperitoneal injection of PRGL493, followed by 50 µl anta-miR-744 intratracheal injection.

ALOX15 Inhibitor + Anta-miR-744 Group: Intraperitoneal injection of ALOX15-IN-2, followed by 50 µl anta-miR-744 intratracheal injection.

GPX4 Inhibitor + miR-NC Group: Intraperitoneal injection of GPX4-IN-3, followed by 50 µl miR-NC intratracheal injection after 60 minutes.

GPX4 Inhibitor + miR-744 Group: Intraperitoneal injection of GPX4-IN-3, followed by 50 µl miR-744 intratracheal injection after 60 minutes.

**Part 2: Assessing the protective effect of miR-744 in LPS-induced ARDS and sepsis.**

Lung Injury Model: Mice received intravenous injections of 50 µL PBS, miR-NC, or miR-744 every 24 hours for three days, followed by a 50 µL(1µg/µL) LPS intratracheal injection to induce lung injury.

Sepsis Model: Mice received intravenous injections of 50 μL PBS, miR-NC, or miR-744 every 24 hours for three days, followed by an intraperitoneal injection of 50 μL(3 μg/μL) LPS to induce sepsis.

24 hours after the final injection, mice were euthanized for organ collection 24 hours post-injection, mice were euthanized for organ collection.

**Part 3: Assessing the effect of anta-miR-744 on epithelial cells *in vivo***

Twenty-four hours after intratracheal injection of anta-miR-744 and miR-NC(both 50µL, 2 µmol/µL), lung tissues were collected for epithelial cell isolation. Lung tissues were dissociated into single-cell suspensions using the Lung Dissociation Kit (Miltenyi Biotec, Cat. No. 130-095-927) following the manufacturer's instructions. The samples were centrifuged at 400g for 5 minutes, red blood cell lysis buffer was added, and then centrifuged again at 400g for 4 minutes. The cells were resuspended in 100 μl of 5% FBS in PBS. The cells were stained with 1 μL CD45 and 2 μL CD326 antibodies, and incubated at 4°C for 40 minutes. After centrifuging at 400g for 5 minutes, the supernatant was discarded, and the cells were resuspended in 300 μl of 5% FBS in PBS. The cells were sorted using flow cytometry. The sorted epithelial cells were used for qPCR, Western blotting, and flow cytometry.

**4: Detailed biochemical analyses**

**qPCR, western blotting, and ELISA**

For qPCR, cells or mouse lung tissues were lysed with RIPA buffer, and RNA was extracted using TRizol. cDNA was synthesized using a Bio-Rad kit and quantified with Maxima™ SYBR Green qPCR Master Mix. mRNA expression was normalized to 18S RNA, and miR-744 to U6. Results were presented as fold changes relative to the miR-744 group. Primers are detailed in **Table S3**. qPCR assessed inflammatory mediator expression in HBE and mouse lungs in response to miRs.

For western blotting, cells and mouse lung samples were collected, and mixed with RIPA buffer, and centrifuged. Total protein concentration was normalized using the BCA Protein Assay kit. Equal amounts of protein samples were separated by sodium dodecyl sulfate-polyacrylamide gel electrophoresis (SDS-PAGE) and transferred to polyvinylidene difluoride (PVDF) membranes which were then blocked with 5% non-fat milk for 1 h at room temperature. The PVDF membranes were incubated at 4 °C overnight with human antibodies against α-tubulin antibody, GPX4 antibody, ACSL4 antibody, ALOX15 antibody. Blots were visualized using HRP-conjugated secondary antibodies and ECL Detection Reagent (Thermo Fisher Scientific) and imaged on a LAS3000 image reader (Bio Rad, UK). The relative amount of protein was quantified using the ratio of α-tubulin.

ELISA was used according to the manufacturer’s instructions to assess MPO in mouse lungs after intratracheal injection of miRs with or without various inhibitors. Concentrations of the mediators were expressed as pg/mL of protein. Data were shown by the fold change normalized to the average value of the sham group.

**H&E and fluorescence colocalization**

Mouse lung samples were collected and fixed in 10% formalin for more than 24 h, dehydrated, paraffin-embedded, and cut into 2-μm-thick sections, which were then deparaffinized and rehydrated.

For H&E staining, the slides were incubated with hematoxylin for five min. After 30 s of dehydration with 100% alcohol, the sections were further treated with eosin for one min. The sections were imaged using a light microscope (Zeiss, Germany) at 200 х magnification. Images were randomly sampled using R software. Lung injury was evaluated using a blinded scoring system focusing on neutrophil presence in alveolar and interstitial spaces, hyaline membranes, proteinaceous debris, and alveolar septal thickening. Each parameter was scored from 0 (none) to 2 (based on severity). Scores were summed and normalized across multiple 400× magnification fields for each of the six mice. H&E staining was performed to evaluate cumulative histopathological changes in mouse lungs.

For fluorescence colocalization analysis, fluorescently labeled miR-744 and anta-miR-744 were administered intratracheally into BALB/c mice. After 2 hours, lung tissues were harvested for preparing fluorescence slides. Slides were incubated with anit-CD326 antibody for 12 h, then incubated with anti-rabbit IgG(H+L) for 2h. Fluorescence colocalization was then examined under an Olympus automated inverted fluorescence microscope (Olympus Corporation, Tokyo, Japan), using specific imaging protocols to identify the localization and interaction of the labeled miRNAs in the lung tissue.
