# Supplementary material for: Reduced microRNA-744 expression in mast cell-derived exosomes triggers epithelial cell ferroptosis in acute respiratory distress syndrome
Source: Redox Biol. 2024 Oct 3;77:103387. doi: 10.1016/j.redox.2024.103387 (PMC11493202; doi:10.1016/j.redox.2024.103387)
Supplement: Multimedia component 2 [file mmc2.pdf]

Figure 1 DE						
**IL6**						
PBS	PBS-CM	LPS-CM	PBS-EXs	LPS-EXs	PBS-EXs-LPS	LPS-EXs-LPS
1.078	1.266	3.766	1.536	5.141	2.277	12.951
0.928	1.451	3	1.527	7.428	2.31	12.614
0.864	1.441	3.029	1.529	4.278	2.505	9.667
0.657	0.662	3.256	1.442	5.614	2.408	7.765
1.522	1.908	3.141	1.385	5.756	2.789	7.89
1.088	1.626	3.061	1.247	4.959	2.491	7.568
**TNFa**						
PBS	PBS-CM	LPS-CM	PBS-EXs	LPS-EXs	PBS-EXs-LPS	LPS-EXs-LPS
0.988	2.184	6.431	2.825	7.873	3.078	18.088
1.013	2.395	4.908	2.645	7.506	3.061	13.881
1.223	2.789	4.281	2.854	4.513	3.173	11.204
0.81	1.497	2.339	1.105	2.928	2.344	4.931
1.235	1.55	2.266	1.127	3.371	2.193	4.853
0.864	1.461	2.083	0.889	3.331	1.989	4.777
**GPX4**						
PBS	PBS-CM	LPS-CM	PBS-EXs	LPS-EXs	PBS-EXs-LPS	LPS-EXs-LPS
1.03	0.844	0.607	0.894	0.508	0.664	0.381
1.033	0.827	0.613	0.9	0.53	0.682	0.436
1.002	0.799	0.59	0.817	0.531	0.676	0.457
0.968	0.792	0.595	0.823	0.558	0.678	0.48
0.978	0.804	0.594	0.821	0.558	0.683	0.479
0.991	0.904	0.61	0.864	0.583	0.682	0.408
**ACSL4**						
PBS	PBS-CM	LPS-CM	PBS-EXs	LPS-EXs	PBS-EXs-LPS	LPS-EXs-LPS
0.998	1.126	2.164	1.126	1.927	2.012	3.172
1.002	1.253	2.118	1.185	1.973	1.816	3.302
1.078	1.246	2.134	1.3	2.063	1.937	3.372
1.037	0.852	2.868	1.015	2.058	1.705	4.26
0.965	0.795	2.449	1.053	2.034	1.487	4.359
0.884	0.791	2.123	1.015	2.278	1.327	4.067
**ALOX15**						
PBS	PBS-CM	LPS-CM	PBS-EXs	LPS-EXs	PBS-EXs-LPS	LPS-EXs-LPS
0.997	0.899	1.48	1.02	1.941	1.852	3.089
1.003	1.296	1.843	1.218	1.952	1.695	3.359
1.225	1.45	1.901	1.699	2.098	2.123	3.411
0.976	0.581	1.937	0.679	1.691	1.452	4.266
1.025	0.531	2.003	0.855	1.731	1.267	4.219
0.907	0.653	1.875	0.643	1.546	1.073	3.742

**Figure 2 H**				
group	GPX4-WT+miR-NC	GPX4-WT+miR-744	GPX4-MUT+miR-NC	GPX4-MUT+miR-744
Luciferase-1	16642	6683	18866	11268
Renilla-1	4694083	3503604	5578234	3385333
Luciferase-2	16524	6517	18458	11254
Renilla-2	4620154	3515716	5542369	3371574
Luciferase-3	16327	6435	18162	11549
Renilla-3	4654258	3616456	5416123	3218159
group	ACSL4-WT+miR-NC	ACSL4-WT+miR-744	ACSL4-MUT+miR-NC	ACSL4-MUT+miR-744
Luciferase-1	32246	15798	25005	20449
Renilla-1	5309816	4184914	4811848	4256030
Luciferase-2	32486	15468	25185	21284
Renilla-2	5347158	4275247	4827954	4217842
Luciferase-3	31714	15316	24927	21845
Renilla-3	5249762	4226861	4852187	4218751
group	ALOX15-WT+miR-NC	ALOX15-WT+miR-744	ALOX15-MUT+miR-NC	ALOX15-MUT+miR-744
Luciferase-1	18885	9486	15851	11035
Renilla-1	5333534	3823711	4817002	3735133
Luciferase-2	18456	9513	15078	11216
Renilla-2	5218842	3836815	4852164	3716632
Luciferase-3	18615	9124	15465	10368
Renilla-3	5287418	3832545	4853722	3732745

Figure 3B	
miR-NC_PBS-MC-EXs	anta-miR-744_PBS-MC-EXs
0.942	0.271
1.061	0.307
1.11	0.306
1.15	0.318
1.426	0.335
1.433	0.347

Figure 3C		
PBS	miR-NC_PBS-MC-EXs	anta-miR-744_PBS-MC-EXs
0.92	1.561	0.492
1.087	2.618	0.628
1.013	2.546	0.608
1.059	2.58	0.639
1.041	2.518	0.639
1.036	2.568	0.801
**Figure 3D**		
GPX4		
PBS	miR-NC_PBS-MC-EXs	anta-miR-744_PBS-MC-EXs
0.953	0.647	0.288
1.05	0.647	0.342
1.082	0.847	0.37
1.103	0.949	0.356
1.069	0.991	0.352
1.135	0.994	0.398
ACSL4		
PBS	miR-NC_PBS-MC-EXs	anta-miR-744_PBS-MC-EXs
0.989	0.953	1.994
1.011	1.018	1.904
0.989	1	1.793
0.995	0.967	1.83
1.08	1.019	1.769
0.988	1.04	1.909
ALOX15		
PBS	miR-NC_PBS-MC-EXs	anta-miR-744_PBS-MC-EXs
1.03	0.893	5.028
0.971	0.99	5.359
0.971	1.061	4.911
1.091	1.061	4.8
1.259	1.107	5.238
1.275	1.097	5.426
IL6		
PBS	miR-NC_PBS-MC-EXs	anta-miR-744_PBS-MC-EXs
1.012	1.491	4.391
0.989	1.607	4.446
1.133	1.467	4.189
1.949	1.718	4.137
2.06	2.429	4.511
2.522	3.02	6.044
TNFa		
PBS	miR-NC_PBS-MC-EXs	anta-miR-744_PBS-MC-EXs
0.804	0.9	2.202
1.244	0.98	2.116
1.578	1.454	2.282
1.796	1.562	2.621
1.724	1.534	2.789
1.701	1.88	3.027

Figure 4B	
miR-NC_LPS-MC-EXs	miR-744_LPS-MC-EXs
0.98	39.138
1.021	38.868
0.997	40.155
1.033	39.988
1.009	40.238
1.003	40.21

Figure 4C		
PBS	miR-NC_LPS-MC-EXs	miR-744_LPS-MC-EXs
1.019	0.491	30.696
0.981	0.496	23.736
1	0.457	27.077
0.876	0.435	29.487
0.642	0.399	28.601
0.57	0.4	30.127
**Figure 4D**		
GPX4		
PBS	miR-NC_LPS-MC-EXs	miR-744_LPS-MC-EXs
0.914	0.343	0.998
1.094	0.346	0.974
1.094	0.337	0.972
1.13	0.345	1.006
1.123	0.329	0.993
0.846	0.321	0.932
ACSL4		
PBS	miR-NC_LPS-MC-EXs	miR-744_LPS-MC-EXs
1.095	1.953	0.698
0.914	1.049	0.881
1.168	1.51	0.888
0.831	2.394	0.96
0.937	1.738	0.931
1.063	2.695	1.626
ALOX15		
PBS	miR-NC_LPS-MC-EXs	miR-744_LPS-MC-EXs
1.415	5.786	0.547
0.707	2.679	0.52
1.153	4.168	0.638
0.777	5.295	0.583
0.821	4.012	0.596
0.613	5.15	0.796
IL6		
PBS	miR-NC_LPS-MC-EXs	miR-744_LPS-MC-EXs
1.295	8.34	0.384
0.772	4.604	0.543
1.26	6.329	0.557
0.76	8.55	0.593
0.845	7.091	0.543
0.875	10.454	0.901
TNFa		
PBS	miR-NC_LPS-MC-EXs	miR-744_LPS-MC-EXs
1.135	13.9	1.677
0.881	6.635	2.817
1.64	12.987	0.893
0.941	13.05	2.56
1.129	11.503	3.171
0.752	12.579	3.683

Figure 5C,D		
GPX4		
PBS	miR-NC+LPS-MC-EXs	miR-744+LPS-MC-EXs
0.879	0.297	0.602
1.138	0.271	0.583
1.454	0.27	0.593
1.474	0.272	0.568
1.448	0.274	0.576
1.274	0.288	0.706
ACSL4		
PBS	miR-NC+LPS-MC-EXs	miR-744+LPS-MC-EXs
1.033	4.117	1.987
0.968	4.283	1.901
0.972	4.443	1.965
0.999	4.361	2.119
1.026	4.382	2.054
0.943	4.3	2.066
ALOX15		
PBS	miR-NC+LPS-MC-EXs	miR-744+LPS-MC-EXs
0.997	5.2	2.306
1.003	6.711	2.482
1.002	6.536	2.622
1.028	6.614	2.618
1.016	6.948	2.712
1.201	7.323	2.71
IL6		
PBS	miR-NC+LPS-MC-EXs	miR-744+LPS-MC-EXs
0.982	4.033	1.791
1.018	3.907	1.968
1.022	3.856	1.975
1.038	3.811	2.022
1.001	3.732	1.962
0.92	3.782	1.994
TNFa		
PBS	miR-NC+LPS-MC-EXs	miR-744+LPS-MC-EXs
0.854	4.838	1.751
1.171	4.719	2.356
1.218	4.693	2.499
1.24	5.361	2.724
1.203	5.73	2.622
1.054	6.895	3.032

miRNA Transfection Figure 6 A				
	PBS	miR-NC	anta-miR-744	miR-744
	1.08584344	0.891214621	0.644927843	25.90591545
	0.84618505	0.94759193	0.679812908	25.58470441
	0.9842083	0.992682249	0.684541381	29.00459683
	0.95172643	1.021959354	0.701837851	29.16587922
	1.05221964	0.98863503	0.69844076	29.32805844
	1.10423595	0.99387493	0.715592246	29.75808246

figure 6D						
PBS	miR-NC	anta-miR-744	ALOX15_IN-anta-miR-744	ACSL4_IN-anta-miR-744	GPX4_IN-miR-NC	GPX4_IN-miR-744
139.723	148.838	85.315	113.834	107.629	66.308	122.373
138.373	145.225	57.533	111.611	101.529	59.02	124.313
143.016	132.53	64.232	117.735	123.426	63.861	126.158

figure 6F. Flow cytometry for ROS		
PBS	miR-NC	anta-miR-744
40	33.1	52.2
40.9	46.2	54.6
44.4	42.6	54.8

figure 6E						
PBS	miR-NC	anta-miR-744	ALOX15_IN-anta-miR-744	ACSL4_IN-anta-miR-744	GPX4_IN-miR-NC	GPX4_IN-miR-744
764	804	1833	1349	1077	2224	1499
778	768	1799	1285	977	2273	1581
830	817	1832	1268	1043	2302	1471
867	848	1785	1295	1042	2293	1533
852	812	1843	1247	1009	2297	1488
835	808	1806	1297	1015	2278	1491

Figure 6H						
GPX4						
PBS	miR-NC	anta-miR-744	ALOX15_IN-anta-miR-744	ACSL4_IN-anta-miR-744	GPX4_IN-miR-NC	GPX4_IN-miR-744
0.94279	1.004864	0.322417	0.778085	0.571173	0.382359	0.65022
1.02243	0.943438	0.354781	0.77164	0.580352	0.366783	0.644387
1.03742	0.953299	0.336342	0.779165	0.591316	0.35233	0.678302
0.97288	0.937788	0.184838	0.588182	0.830086	0.364334	0.609768
1.03192	0.937788	0.199759	0.537871	0.76755	0.356095	0.598878
0.99608	0.886587	0.191888	0.506044	0.814131	0.337354	0.611885
ACSL4						
PBS	miR-NC	anta-miR-744	ALOX15_IN-anta-miR-744	ACSL4_IN-anta-miR-744	GPX4_IN-miR-NC	GPX4_IN-miR-744
0.96571	1.044394	2.377865	1.97201	1.41095	2.915345	1.886440591
0.98328	0.984639	2.50475	1.762553	1.443596	2.516933	1.610676394
1.05312	0.983275	2.582318	1.700155	1.444597	2.550299	1.837401188
1.08448	0.87904	2.577549	1.689973	1.118062	2.602684	1.984808749
0.91067	0.843816	2.579337	1.669019	1.354725	2.321408	1.831467373
1.01256	0.852635	2.503593	1.540074	1.270151	2.353813	1.888184838
ALOX15						
PBS	miR-NC	anta-miR-744	ALOX15_IN-anta-miR-744	ACSL4_IN-anta-miR-744	GPX4_IN-miR-NC	GPX4_IN-miR-744
0.90063	1.178539	5.61778	1.404445	2.005553	4.76673	1.823866331
0.87782	1.185093	4.594793	1.497961	1.36604	4.563055	1.851892045
1.26488	0.963262	3.994459	1.482467	1.278099	3.877159	1.863480859
0.85145	0.641713	3.893317	1.251796	1.151089	3.267076	1.825130977
0.84851	0.617709	3.89062	1.274561	1.128182	2.838247	1.609932275
1.38415	0.635516	3.703784	1.387031	1.104199	2.775985	1.606587994

**Figure7 A**						
0h						
PBS	miR-NC	anta-miR-744	ALOX15_IN-anta-miR-744	ACSL4_IN-anta-miR-744	GPX4_IN-miR-NC	GPX4_IN-miR-744
0.418	0.425	0.415	0.423	0.417	0.426	0.425
0.422	0.412	0.417	0.421	0.416	0.422	0.418
0.423	0.416	0.423	0.417	0.424	0.414	0.423
0.426	0.425	0.422	0.419	0.423	0.415	0.426
0.412	0.427	0.416	0.424	0.413	0.422	0.412
0.417	0.415	0.425	0.414	0.427	0.414	0.423
24h						
PBS	miR-NC	anta-miR-744	ALOX15_IN-anta-miR-744	ACSL4_IN-anta-miR-744	GPX4_IN-miR-NC	GPX4_IN-miR-744
0.435	0.443	0.421	0.428	0.424	0.422	0.429
0.446	0.435	0.426	0.432	0.422	0.426	0.431
0.442	0.438	0.418	0.425	0.432	0.417	0.427
0.445	0.448	0.423	0.427	0.427	0.423	0.435
0.431	0.447	0.415	0.435	0.418	0.428	0.426
0.437	0.434	0.427	0.423	0.433	0.421	0.434
48h						
PBS	miR-NC	anta-miR-744	ALOX15_IN-anta-miR-744	ACSL4_IN-anta-miR-744	GPX4_IN-miR-NC	GPX4_IN-miR-744
0.517	0.523	0.446	0.472	0.462	0.441	0.465
0.526	0.518	0.443	0.468	0.465	0.438	0.467
0.515	0.522	0.452	0.466	0.476	0.445	0.462
0.521	0.516	0.453	0.475	0.471	0.442	0.451
0.516	0.524	0.447	0.465	0.467	0.432	0.453
0.514	0.513	0.441	0.467	0.473	0.436	0.442
72h						
PBS	miR-NC	anta-miR-744	ALOX15_IN-anta-miR-744	ACSL4_IN-anta-miR-744	GPX4_IN-miR-NC	GPX4_IN-miR-744
0.651	0.648	0.492	0.537	0.543	0.482	0.535
0.657	0.652	0.488	0.546	0.546	0.476	0.537
0.648	0.654	0.486	0.542	0.536	0.472	0.528
0.643	0.642	0.495	0.536	0.538	0.486	0.531
0.656	0.647	0.496	0.548	0.547	0.483	0.533
0.655	0.653	0.483	0.545	0.542	0.475	0.526

**Figure7 E**						
IL6						
PBS	miR-NC	anta-miR-744	ALOX15_IN-anta-miR-744	ACSL4_IN-anta-miR-744	GPX4_IN-miR-NC	GPX4_IN-miR-744
0.84557	1.16635	3.855719	1.879045	1.58447	3.97237	1.957483301
1.07923	1.09809	3.800002	2.009728	1.719513	3.312683	1.705269784
1.09581	1.10191	3.545527	1.896054	1.717131	3.630077	1.73868912
0.6043	0.59063	2.031201	0.985094	0.83586	1.966095	1.092272885
0.62518	0.5611	1.979771	0.969514	0.804594	1.797926	0.935191248
0.64231	0.53601	1.926969	0.910249	0.817524	1.756049	0.987829072
TNFa						
PBS	miR-NC	anta-miR-744	ALOX15_IN-anta-miR-744	ACSL4_IN-anta-miR-744	GPX4_IN-miR-NC	GPX4_IN-miR-744
0.73816	1.42504	2.56152	1.689973	1.776454	3.575141	1.73628
1.35473	1.53901	2.752991	2.121375	1.461044	2.911979	1.892115
1.88165	1.3491	2.728296	1.979313	1.922522	2.662903	2.016705
1.04114	0.88281	1.631463	1.246745	1.275592	3.099782	1.205112
1.00498	0.89265	1.631463	1.133015	1.24761	2.866254	1.063018
0.9132	0.83973	2.803055	1.86995	1.611049	3.319578	2.323017
1.09505	1.06807	3.448574	2.050534	1.607702	3.344986	2.443586

miRNA intratracheal administration(Figure 8 A)			
PBS	miR-NC	MIR744	anta-miR-744
0.81	1.04	20.64	0.55
0.95	1.16	18.70	0.57
1.08	1.08	18.75	0.58
1.06	1.10	16.62	0.60
1.05	1.10	17.09	0.61
1.08	1.12	16.83	0.60

qPCR for Figure 8H,J

GPX4	
miR-NC	anta-miR-744
0.91	0.22
1.10	0.22
1.12	0.30
1.15	0.33
1.51	0.39
1.55	0.56
0.85	0.31

ACSL4	
miR-NC	anta-miR-744
1.00	1.11
1.00	1.19
1.01	1.16
1.01	1.22
1.01	1.24
1.05	1.20

ALOX15	
miR-NC	anta-miR-744
0.94	1.60
1.06	1.67
1.06	2.41
1.27	2.92
1.73	2.98
1.72	2.93

IL6	
miR-NC	anta-miR-744
1.01	3.61
0.99	4.32
1.01	4.08
0.99	4.10
1.07	4.30
1.12	4.40

TNFa	
miR-NC	anta-miR-744
0.94	1.22
1.07	1.30
1.07	1.26
1.08	1.29
1.19	1.31
1.22	1.38

MPO(pg/mL) for Figure 8F						
PBS	miR-NC	anta-miR-744	ALOX15_IN-anta-miR-744	ACSL4_IN-anta-miR-744	GPX4_IN-miR-NC	GPX4_IN-miR-744
64.80	55.92	80.02	66.70	76.85	110.47	89.53
67.34	60.99	87.00	73.04	74.95	113.00	87.63
62.26	58.46	83.83	70.51	77.48	108.56	85.09
54.65	61.63	92.07	75.58	73.68	100.32	80.66
49.58	65.43	87.63	74.31	71.78	104.12	76.85
54.02	66.70	89.53	77.48	74.31	102.22	80.02

lung injury scores for Figure 8E						
PBS	miR-NC	anta-miR-744	ACSL4_IN-anta-miR-744	ALOX15_IN-anta-miR-744	GPX4_IN-miR-NC	GPX4_IN-miR-744
0.28	0.33	0.64	0.43	0.28	0.73	0.51
0.28	0.33	0.67	0.45	0.41	0.72	0.48
0.33	0.28	0.80	0.37	0.39	0.78	0.50
0.28	0.28	0.68	0.28	0.30	0.73	0.38
0.33	0.38	0.70	0.38	0.35	0.76	0.39
0.38	0.40	0.70	0.40	0.36	0.75	0.39

Figure 9 B,C

IL6

PBS	miR-NC	LPS	anta-miR-744+LPS	miR-NC+LPS	miR-744+LPS
0.99	1.06	2.33	3.56	2.84	2.08
1.01	1.07	2.52	4.03	2.80	2.01
1.00	1.09	2.48	4.09	2.95	2.05
1.11	0.99	3.21	4.81	3.58	2.53
1.09	1.05	3.06	4.94	3.30	2.20
0.83	1.02	2.76	4.51	3.29	2.26

TNFa

PBS	miR-NC	LPS	anta-miR-744+LPS	miR-NC+LPS	miR-744+LPS
1.04	1.11	2.10	4.65	2.28	1.35
1.03	1.10	2.01	4.52	2.32	1.31
0.94	1.07	1.77	4.00	2.35	1.41
0.93	1.04	2.16	3.63	2.23	1.90
1.07	1.19	2.45	5.98	2.34	1.91

GPX4

PBS	miR-NC	LPS	anta-miR-744+LPS	miR-NC+LPS	miR-744+LPS
0.97	1.14	0.56	0.38	0.64	0.94
1.02	1.12	0.60	0.38	0.72	0.89
1.01	1.14	0.61	0.39	0.73	0.88
1.01	0.99	0.64	0.39	0.61	0.72
1.07	1.05	0.59	0.36	0.63	0.69
0.92	0.99	0.60	0.36	0.64	0.73

ACSL4

PBS	miR-NC	LPS	anta-miR-744+LPS	miR-NC+LPS	miR-744+LPS
0.98	1.03	2.01	3.15	2.39	1.88
1.01	1.24	2.10	3.20	2.45	1.86
1.01	1.27	2.24	3.22	2.60	1.79
1.07	1.04	3.19	4.95	3.22	2.15
1.10	1.05	2.87	4.44	3.21	1.89
0.85	1.06	2.65	4.01	3.49	1.92

ALOX15

PBS	miR-NC	LPS	anta-miR-744+LPS	miR-NC+LPS	miR-744+LPS
0.95	1.13	2.00	2.25	2.08	1.48
0.94	1.19	1.93	2.95	2.08	1.51
1.04	1.18	1.83	2.98	2.07	1.71
1.01	1.19	1.76	2.93	2.08	1.71
1.05	1.17	1.75	3.33	2.16	1.77
1.02	1.17	1.78	3.37	2.21	1.74

Figure9 EF

IL6

Sham	LPS	miR-NC+LPS	miR-744+LPS
1.06	1.41	2.96	1.35
0.94	1.78	3.03	1.26
0.92	1.55	1.22	1.19
1.09	1.45	1.59	1.14
0.95	2.51	2.70	1.42
1.05	2.88	2.90	1.67

TNFa

Sham	LPS	miR-NC+LPS	miR-744+LPS
1.06	1.41	2.96	1.35
0.94	1.78	3.03	1.26
0.92	1.55	1.22	1.19
1.09	1.45	1.59	1.14
0.95	2.51	2.70	1.42
1.05	2.88	2.90	1.67

GPX4

Sham	LPS	miR-NC+LPS	miR-744+LPS
1.06	0.36	0.36	0.80
0.95	0.38	0.35	0.82
0.99	0.44	0.35	0.64
1.01	0.45	0.40	0.63
0.99	0.37	0.35	0.41
1.01	0.39	0.36	0.56

ACSL4

Sham	LPS	miR-NC+LPS	miR-744+LPS
1.00	2.37	2.92	1.36
1.00	2.81	3.07	1.33
1.07	2.18	1.77	1.39
0.93	2.29	1.95	1.39
1.00	2.16	2.07	0.81
1.00	2.32	2.15	1.07

ALOX15

Sham	LPS	miR-NC+LPS	miR-744+LPS
0.97	1.61	1.80	1.06
1.03	1.78	2.05	1.22
0.93	1.64	1.68	0.78
1.07	1.71	1.77	0.99
0.95	1.38	1.41	1.29
1.05	1.52	1.57	1.32

Figure S5

IL6

Sham	LPS	miR-NC+LPS	miR-744+LPS
0.97	1.97	1.50	1.10
1.03	1.82	1.55	1.23
0.89	2.79	2.23	1.75
1.12	2.50	2.19	1.83
0.61	1.52	1.90	0.96
0.63	1.70	1.80	0.96

TNFa

Sham	LPS	miR-NC+LPS	miR-744+LPS
0.99	1.40	1.55	1.06
1.01	1.59	1.62	1.13
0.91	1.39	1.63	1.12
1.10	1.41	1.75	1.28
0.87	1.88	1.42	0.87
1.15	1.86	1.49	1.06

GPX4

Sham	LPS	miR-NC+LPS	miR-744+LPS
0.99	0.55	0.58	0.73
1.02	0.65	0.62	0.76
1.00	0.36	0.40	0.67
1.00	0.35	0.41	0.68
1.01	0.52	0.36	0.63
0.99	0.45	0.36	0.66

ACSL4

Sham	LPS	miR-NC+LPS	miR-744+LPS
0.93	2.64	2.58	1.39
1.08	2.83	2.72	1.55
0.93	2.17	1.83	1.02
1.08	2.09	1.67	1.14
0.94	1.91	1.97	0.99
1.07	1.99	2.15	1.04

ALOX15

Sham	LPS	miR-NC+LPS	miR-744+LPS
0.93	1.82	1.93	1.16
1.07	1.89	1.99	1.21
1.01	1.45	1.45	0.82
0.99	1.60	1.40	0.99
0.89	1.84	1.93	1.18
1.12	1.98	2.04	1.63

Figure10A	
PBS	LPS
0.95247293	0.57784321
1.03580326	0.54064369
1.00538635	0.53952062
1.00817774	0.5413937
0.99705846	0.54327328
0.93740864	0.54176909
0.93416545	0.54064369
0.96309499	0.4988749
0.99427627	0.37600498
1.00575667	0.37950469
0.9091521	0.3524649
1.05304009	0.36377194

Figure 10B			
blood		Exosome	
PBS	ARDS	PBS	ARDS
1.0895	0.03963	1.01256	0.57995
0.91362	0.03752	0.97603	0.56448
1.00463	0.03621	1.01185	0.53849
1.30285	0.16977	1.10343	0.40304
1.28669	0.27694	1.01256	0.41123
1.16044	0.28003	1.03455	0.52522
2.27469	0.04277	1.2861	0.55402
2.27469	0.04782	1.30496	0.52123
2.11795	0.04716	1.26313	0.51548
1.09	0.11561	1.44193	0.52888
0.9555	0.11245	1.37745	0.537
0.96015	0.11098	1.14235	0.44566
0.29696	0.10506	2.20381	0.63994
0.30446	0.1072	2.27363	0.63949
0.30006	0.10085	2.30537	0.65429
2.09701	0.02525	2.35381	0.72951
2.28998	0.02389	2.21453	0.7495
2.3075	0.02307	2.17799	0.68492

FigS3.B,CD			
GPX4			
WT	MC-/-	LPS+WT	LPS+MC-/-
0.92	1.04	0.24	0.54
1.08	1.05	0.28	0.61
0.98	0.93	0.31	0.55
1.00	0.99	0.39	0.58
0.93	1.19	0.41	0.65
1.08	1.08	0.40	0.69
ACSL4			
WT	MC-/-	LPS+WT	LPS+MC-/-
0.91	0.87	4.05	2.13
1.10	0.95	5.08	2.66
1.03	0.87	5.35	2.97
1.31	0.97	5.38	3.46
1.01	1.10	8.42	5.11
0.99	1.13	9.74	3.76
ALOX15			
WT	MC-/-	LPS+WT	LPS+MC-/-
0.98	1.06	5.53	3.31
1.02	1.05	5.68	3.51
1.00	0.94	5.56	3.39
1.03	0.96	5.65	3.28
1.02	1.39	4.06	1.96
0.98	1.50	5.54	1.66
IL6			
WT	MC-/-	LPS+WT	LPS+MC-/-
0.99	0.95	4.87	3.15
1.01	1.01	5.19	3.15
0.99	0.89	5.21	2.77
0.96	0.92	5.04	2.86
1.01	0.88	3.84	1.95
0.99	0.90	4.47	1.51
TNFa			
WT	MC-/-	LPS+WT	LPS+MC-/-
1.06	0.87	2.99	1.82
0.95	0.80	2.93	1.94
0.97	0.82	3.25	2.06
0.87	0.82	3.26	1.66
0.98	1.29	4.61	2.17
1.02	1.16	4.09	2.32
miR-744			
WT	MC-/-	LPS+WT	LPS+MC-/-
1.03	0.73	0.12	0.56
0.97	0.81	0.13	0.60
0.91	0.89	0.17	0.59
0.96	0.89	0.21	0.61
0.92	0.80	0.07	0.42
1.20	0.73	0.07	0.43

FigS3.E,F.G			
GPX4(ng/mL)			
WT	MC-/-	LPS+WT	LPS+MC-/-
11.74	10.97	4.44	7.02
16.31	10.62	4.85	7.13
12.19	12.11	1.89	4.37
14.40	11.27	1.61	4.51
15.31	13.65	4.50	6.00
16.67	13.83	4.92	5.92
ACSL4(ng/mL)			
WT	MC-/-	LPS+WT	LPS+MC-/-
4.50	13.00	56.68	25.51
4.62	10.17	46.50	26.58
9.35	5.57	43.77	31.10
8.65	6.60	42.17	34.76
3.51	9.68	49.53	30.09
3.88	7.50	48.95	29.09
ALOX15(ng/mL)			
WT	MC-/-	LPS+WT	LPS+MC-/-
0.83	0.91	4.44	2.46
0.69	1.33	3.69	2.65
0.97	0.71	3.83	2.52
0.86	0.62	3.71	2.75
0.86	0.90	3.42	2.30
0.90	1.10	3.52	2.91
IL6(pg/mL)			
WT	MC-/-	LPS+WT	LPS+MC-/-
8.71	9.92	15.37	12.15
8.87	10.58	15.52	13.40
8.97	10.45	15.90	13.55
9.99	9.76	15.15	13.10
8.12	9.99	15.68	11.83
8.82	9.89	15.28	12.87
TNFa(pg/mL)			
WT	MC-/-	LPS+WT	LPS+MC-/-
36.49	39.62	60.33	51.15
32.05	40.39	62.53	46.01
33.48	34.62	66.58	47.48
29.22	35.87	61.43	48.58
34.99	41.56	70.62	53.79
38.81	36.09	68.78	56.77
MPO(pg/mL)			
WT	MC-/-	LPS+WT	LPS+MC-/-
256.38	256.38	397.63	344.66
249.76	273.38	397.63	339.15
276.24	278.89	375.56	324.80
306.26	273.60	383.29	322.59
256.38	272.71	366.73	343.56
219.96	266.75	367.84	331.31

qPCR for Figure S4 A,B						
GPX4						
PBS	miR-NC	anta-miR-744	ALOX15_IN-anta-miR-744	ACSL4_IN-anta-miR-744	GPX4_IN-miR-NC	GPX4_IN-miR-744
1.01	0.95	0.50	0.80	0.75	0.51	0.87
0.94	0.94	0.53	0.81	0.73	0.51	0.84
1.06	1.03	0.53	0.96	0.84	0.62	0.80
1.07	1.10	0.46	0.97	0.76	0.61	0.66
0.93	1.17	0.47	0.92	0.77	0.65	0.86
0.99	0.93	0.26	0.66	0.69	0.43	0.88
1.01	0.85	0.31	0.74	0.80	0.62	0.87
ACSL4						
PBS	miR-NC	anta-miR-744	ALOX15_IN-anta-miR-744	ACSL4_IN-anta-miR-744	GPX4_IN-miR-NC	GPX4_IN-miR-744
1.02	0.69	2.84	1.66	1.39	2.23	0.53
0.96	0.77	2.94	1.48	1.38	1.98	0.53
1.02	0.86	3.00	1.64	1.44	1.91	0.59
1.05	0.83	2.56	1.30	1.21	1.36	1.29
0.96	0.90	2.58	1.28	1.27	1.80	1.44
0.98	0.90	2.41	1.36	1.38	1.77	1.38
ALOX15						
PBS	miR-NC	anta-miR-744	ALOX15_IN-anta-miR-744	ACSL4_IN-anta-miR-744	GPX4_IN-miR-NC	GPX4_IN-miR-744
0.92	0.78	1.23	0.66	0.96	2.01	1.12
0.91	0.94	1.76	1.04	0.94	2.04	1.16
1.20	1.00	1.99	1.17	1.36	2.19	1.26
1.02	0.80	2.71	1.29	1.54	3.08	1.50
0.98	0.73	2.66	1.46	1.41	3.03	1.58
IL6						
PBS	miR-NC	anta-miR-744	ALOX15_IN-anta-miR-744	ACSL4_IN-anta-miR-744	GPX4_IN-miR-NC	GPX4_IN-miR-744
0.38	0.47	0.88	0.79	0.64	0.96	0.52
0.39	0.45	1.00	0.82	0.68	0.94	0.52
0.38	0.46	1.11	0.82	0.69	0.95	0.53
0.64	0.70	1.00	0.64	0.81	1.05	0.70
0.61	0.70	0.98	0.64	0.84	1.02	0.69
0.59	0.71	1.02	0.62	0.91	1.03	0.67
TNFa						
PBS	miR-NC	anta-miR-744	ALOX15_IN-anta-miR-744	ACSL4_IN-anta-miR-744	GPX4_IN-miR-NC	GPX4_IN-miR-744
0.64	0.70	1.00	0.64	0.81	1.05	0.70
0.61	0.70	0.98	0.64	0.84	1.02	0.69
0.59	0.71	1.02	0.62	0.91	1.03	0.67
0.31	0.21	0.87	0.64	0.46	0.82	0.48
0.38	0.31	0.95	0.65	0.69	0.81	0.48
0.37	0.31	1.18	0.69	0.75	0.89	0.53
